# Creation and Initial Characterization of Isogenic *Helicobacter pylori* CagA EPIYA Variants Reveals Differential Activation of Host Cell Signaling Pathways

**DOI:** 10.1038/s41598-017-11382-y

**Published:** 2017-09-08

**Authors:** Dacie R. Bridge, Faith C. Blum, Sungil Jang, Jinmoon Kim, Jeong-Heon Cha, D. Scott Merrell

**Affiliations:** 1 Uniformed Services University of the Health Sciences, F. Edward Hébert School of Medicine, Department of Microbiology and Immunology, Bethesda, Maryland 20814 USA; 2 Department of Oral Biology, Oral Science Research Center, Yonsei University College of Dentistry, Seoul, South Korea; 3 Department of Applied Life Science, BK21 Plus Project, Yonsei University College of Dentistry, Seoul, South Korea; 4 Microbiology & Molecular Biology, Key Laboratory of Oral Medicine, Guangzhou Institute of Oral Disease, Stomatology Hospital of Guangzhou Medical University, Guangzhou, China; 5 Present Address: University of Maryland School of Medicine, Center for Vaccine Development, Division of Geographic Medicine, Department of Medicine, Baltimore Maryland, 21201 USA

## Abstract

The polymorphic CagA toxin is associated with *Helicobacter pylori*-induced disease. Previous data generated using non-isogenic strains and transfection models suggest that variation surrounding the C-terminal Glu-Pro-Ile-Tyr-Ala (EPIYA) motifs as well as the number of EPIYA motifs influence disease outcome. To investigate potential CagA-mediated effects on host cell signaling, we constructed and characterized a large panel of isogenic *H. pylori* strains that differ primarily in the CagA EPIYA region. The number of EPIYA-C motifs or the presence of an EPIYA-D motif impacted early changes in host cell elongation; however, the degree of elongation was comparable across all strains at later time points. In contrast, the strain carrying the EPIYA-D motif induced more IL-8 secretion than any other EPIYA type, and a single EPIYA-C motif induced comparable IL-8 secretion as isolates carrying multiple EPIYA-C alleles. Similar levels of ERK1/2 activation were induced by all strains carrying a functional CagA allele. Together, our data suggest that polymorphism in the CagA C-terminus is responsible for differential alterations in some, but not all, host cell signaling pathways. Notably, our results differ from non-isogenic strain studies, thus highlighting the importance of using isogenic strains to study the role of CagA toxin polymorphism in gastric cancer development.

## Introduction

*Helicobacter pylori* is causally associated with gastric and duodenal ulcers, gastric cancer, and MALT-lymphoma in approximately 20% of infected individuals^1–4^. Despite the identification of *H. pylori*’s role in gastric cancer development and classification of the bacterium as a class I carcinogen^5^, gastric carcinoma remains the third leading cause of cancer-related mortality^6^. *H. pylori*-associated disease development is a multifactorial process where, in addition to host, diet, and environmental factors^7^, *H. pylori* virulence factors contribute to disease progression; among these are the *cag*-pathogenicity island (*cag*-PAI)^8^, cytotoxin-associated gene A (CagA), vacuolating toxin A (VacA), and numerous outer membrane proteins^9^. The *cag*-PAI is carried by the majority of *H. pylori* strains and encodes a type IV secretion system (T4SS) that is responsible for CagA translocation directly into host cells^8^. *H. pylori* strains encoding the *cag-*PAI and CagA are more virulent^8, 9^.

CagA was defined as an oncoprotein due to its ability to induce gastric polyps and adenocarcinoma when transgenically expressed in mice^10^. Extensive *in vitro* studies have shown that following translocation into host cells, CagA binds to the inner leaflet of the host cell plasma membrane and is phosphorylated by c-Src and c-Abl at tyrosine residues located in the C-terminal Glu-Pro-Ile-Tyr-Ala (EPIYA) sequence of the protein^11–16^. Phosphorylated CagA then induces aberrant host cell signaling through formation of a complex with more than 20 host cell proteins^17^, chief among these is SHP-2^18, 19^. Subsequent host cell signaling pathway stimulation results in host cell elongation termed the “hummingbird phenotype”^20^, which is characterized by loss of host cell polarity and tight junction formation, cytoskeletal rearrangements, and increased host cell motility^21–24^. CagA also activates proteins that increase host cell proliferation and aberrant host cell survival^25, 26^, and induces host inflammatory responses primarily through modulation of nuclear factor-κB (NF-κB)^27, 28^. Together the ability of *H. pylori* ^29–31^ and CagA^32, 33^ to deregulate these host cell processes contributes to epithelial-to-mesenchymal transition (EMT)^34^, which in turn may contribute to gastric carcinogenesis^9^.

A significant factor in host cell pathway modulation is allelic variation in the CagA C-terminal EPIYA motifs^35^; the number of EPIYA repeats and the amino acid sequences that surround the repeats vary across *H. pylori* strains^36^. As such, strains originating from the United States, Europe, and Australia contain EPIYA-A, -B, and -C alleles, while strains originating from Japan, South Korea, or China contain EPIYA-A, -B, and -D alleles^37, 38^. The timing and degree of CagA phosphorylation appears to be dependent on the EPIYA motif that is present. In this regard, EPIYA-C and -D motifs are preferentially phosphorylated by c-Src early in infection, whereas c-Abl can phosphorylate all four motifs at later time points^39^. However, despite the importance of CagA in disease progression, it remains unclear how variation in the EPIYA region of the toxin ultimately affects various host cell signaling pathways. While numerous studies have sought to define the importance of CagA polymorphism in host cell changes^24, 35, 40–42^, most of that work was conducted using non-isogenic strains or transfection assays. Therefore, those results may not recapitulate what would occur during a natural infection or may be influenced by *H. pylori*’s high rate of genetic variability^9^. To date a single collection of isogenic strains (CagA EPIYA-AB, -ABC, and -ABCCC alleles) has been constructed to study the role of the CagA EPIYA motif variation in host cell signaling^33, 43, 44^. Of note, the EPIYA-ABD form of the toxin is significantly associated with gastric cancer development^41^; it is believed to activate signaling pathways more efficiently than a single EPIYA-C allele due to a stronger binding interaction with SHP-2^35^. Therefore, the absence of the CagA EPIYA-ABD allele in the aforementioned isogenic strain panel may prevent identification of pathways altered solely by the EPIYA-D motif. Furthermore, strains containing additional EPIYA-C combinations are found in nature^45^.

To expand our understanding of the significance of CagA polymorphism, we developed a set of *H. pylori* isogenic strains that differ primarily in the CagA EPIYA region. Though not exhaustive, our collection was designed to encompass some of the most commonly found CagA variants seen in *H. pylori* clinical isolates. Herein, we describe the construction and preliminary characterization of a *H. pylori* G27 isogenic strain collection that contains EPIYA-AB, -ABC, -ABCC, -ABCCC, -ABCCCC, and -ABD alleles of the CagA toxin. As additional comparators, we constructed isogenic strains that were deficient in CagA (Δ*cagA*), contained a deletion of the CagA EPIYA region (ΔEPIYA), were lacking the *cag*-PAI (ΔPAI), contained an insertion mutation leading to expression of just a portion of the CagA N-terminus (*cagA::cat*), or were restored to the G27 wild type (WT) EPIYA type (ABCC Restorant). Characterization of the isogenic strain panel suggests CagA EPIYA-dependent differential regulation of some host cell signaling pathways. Thus, our study highlights the importance of using isogenic strains to characterize differences in CagA EPIYA-dependent induction of host cell signaling.

## Results

### Construction and basic characterization of isogenic *H. pylori* strains that differ primarily in the CagA EPIYA region

**Figure 1:**
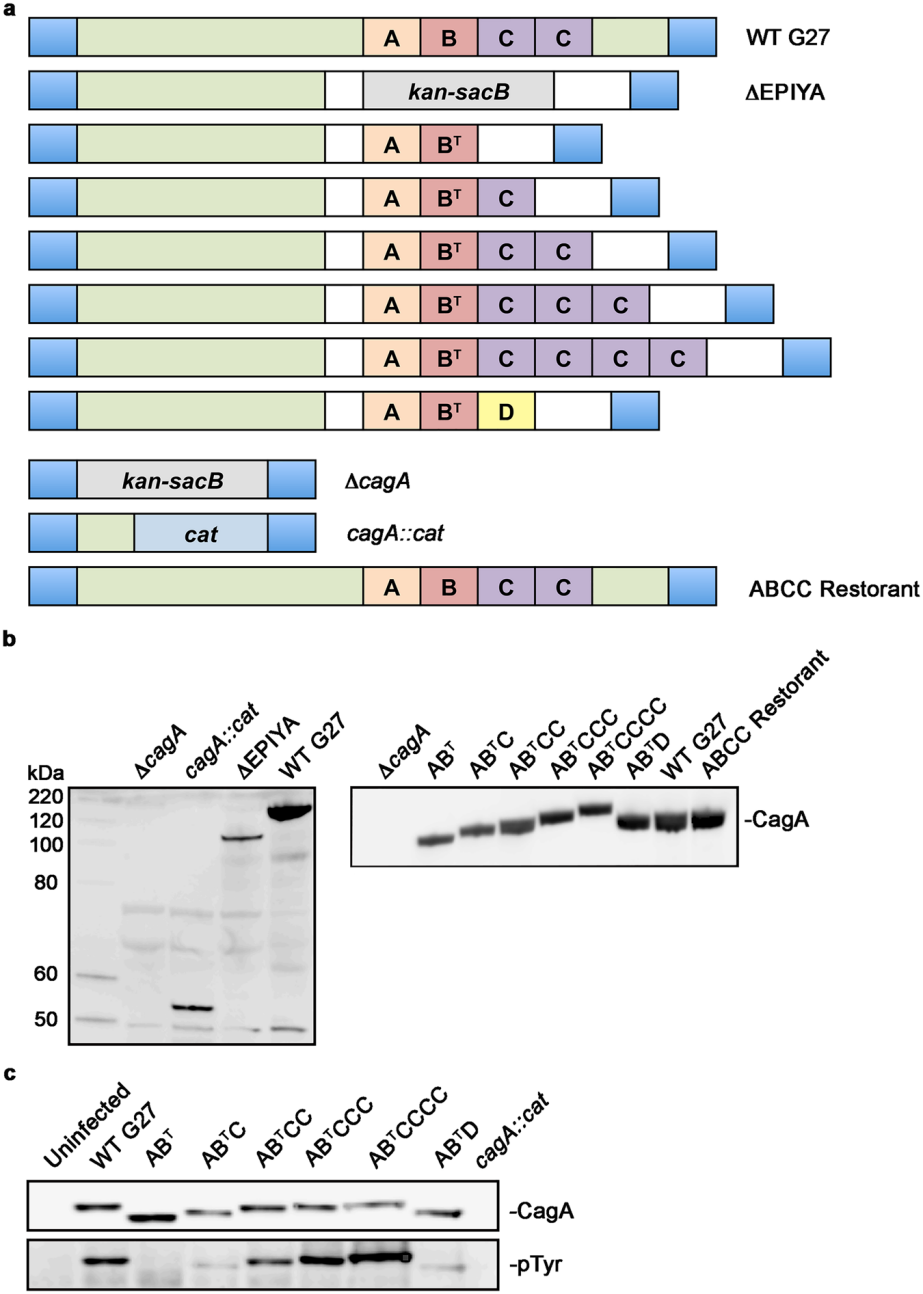
Construction and characterization of the G27 CagA isogenic strains. (**a**) *H. pylori* G27 (CagA EPIYA-ABCC) was used to construct isogenic strains that differ primarily in the form of CagA expressed. The C-terminal EPIYA region of WT G27 was replaced with the counter-selectable *kan-sacB* cassette (gray box) to create the ΔEPIYA strain. The EPIYA-AB^T^, -AB^T^C, -AB^T^CC, -AB^T^CCC, -AB^T^CCCC motifs were PCR amplified from clinical isolate K154, while the EPIYA-AB^T^D was amplified from the clinical isolate K3. Of note, as the EPIYA-B motif of K154 contains a natural alanine to threonine mutation (denoted as B^T^), the EPIYA-B motif alanine of EPIYA-ABD from K3 was mutated to EPIYT. The ΔEPIYA strain was naturally transformed with vectors carrying the various EPIYA constructs and the *kan-sacB* cassette was replaced via double homologous recombination. As controls, the entire *cagA* coding sequence was replaced with a *kan-sacB* cassette (Δ*cagA*), a truncated CagA protein was constructed with the addition of a *cat* cassette (light blue box; *cagA::cat*), and as a control for genetic manipulation, the ΔEPIYA strain was restored to its WT genotype (ABCC Restorant). Blue-G27 genomic DNA sequence; Green-G27 *cagA* sequence; White-7.13 flanking sequence; Orange-EPIYA-A motif; Red-EPIYA B motif; Purple-EPIYA-C motifs; Yellow-EPIYA-D motif; (**b**). The isogenic strains were analyzed for proper deletion, truncation, or expression of CagA by Western blot. For the image on the right, the Western blot image was cropped to show only the region corresponding to CagA (**c**). AGS cells were infected with each isogenic strain at an MOI of 100 for 8 hrs and whole cell lysates were analyzed by Western blot using anti-CagA and anti-phosphotyrosine antibodies to detect total (CagA) and phosphorylated CagA (pTyr). The Western blot images were cropped to show only the region corresponding to CagA (top) and phosphorylated CagA (bottom). Together, the panel of isogenic strains was able to secrete CagA that could be translocated and phosphorylated in AGS cells.

Previous studies indicate that variation in the CagA EPIYA region contributes to differences in disease progression^23, 34, 39–41^. However, those studies were largely completed utilizing transfection models or non-isogenic strains. Thus, the previous results may not recapitulate what occurs during a natural infection or solely depend on CagA, respectively. To this end, we created *H. pylori* isogenic strains in G27 that differ primarily in the CagA EPIYA region (Supplementary Figure S1) and encompass the following variants: EPIYA-AB^T^, -AB^T^C, -AB^T^CC, -AB^T^CCC, -AB^T^CCCC and -AB^T^D (Fig. 1a). The following control strains were created for comparison purposes: Δ*cagA*, *cagA::cat*, ΔEPIYA, and a G27 strain restored to the WT ABCC genotype (Fig. 1a). Thus, a total of 10 isogenic strains were created and characterized alongside WT G27.

Western blot analysis revealed that the size of the CagA protein expressed from each of the isogenic strains reflected the expected size of the protein based on the number of EPIYA motifs (Fig. 1b). Moreover, each of the control strains showed the expected loss or change in CagA size compared to WT G27 (Fig. 1b). Characterization of the *in vitro* growth characteristics of the isogenic strains revealed growth kinetics that virtually mirrored the parental G27 strain (Supplementary Fig. S2). Moreover, EPIYA variation appeared to play no significant role in bacterial adherence to or internalization into AGS cells (Supplementary Fig. S2). To ensure that each of the strains retained the ability to translocate CagA into host cells, whole cell lysates of infected AGS cells were analyzed by Western blot using anti-CagA and anti-phosphotyrosine antibodies. While equivalent levels of CagA were expressed by each strain, varying levels of phosphorylated CagA were detected for each isogenic strain (Fig. 1c). No phosphorylated band was detected for the uninfected cells or the *cagA::cat*, ΔEPIYA, or Δ*cagA* controls (Fig. 1c and Supplementary Fig. S2). Additionally, CagA translocation and phosphorylation by the ABCC Restorant was comparable to WT G27 (Supplementary Fig. S2). Taken together, these data suggest that the isogenic strain collection may be a useful tool to study downstream effects of translocation and phosphorylation of the various CagA variants.

### Effect of EPIYA variation on host cell elongation

**Figure 2:**
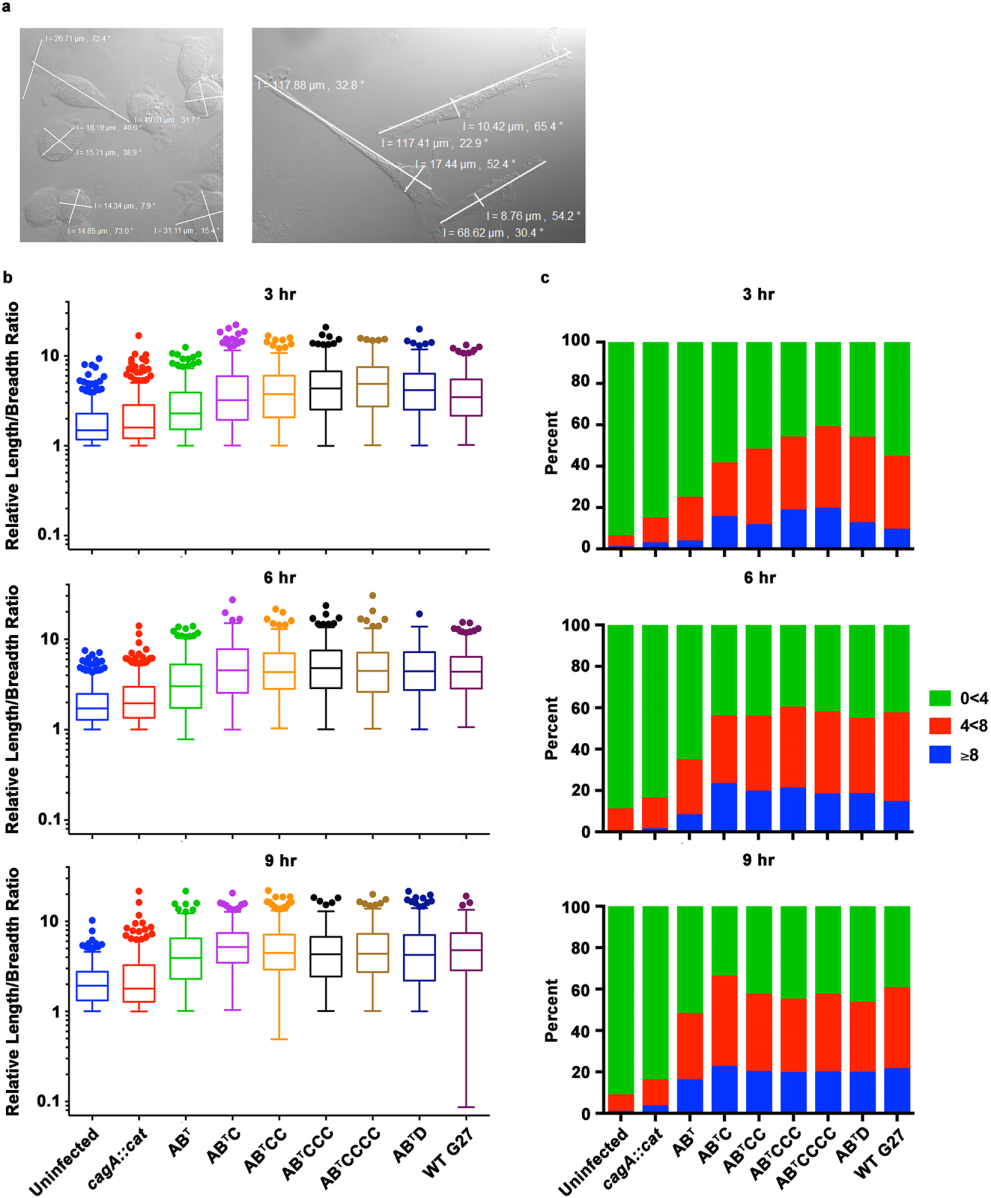
CagA EPIYA-C and -D motifs affect the timing but not the ultimate overall level of AGS cell elongation. AGS cells were infected with the isogenic strains at an MOI of 10 for 3, 6, or 9 hrs. The cells were fixed and imaged by DIC microscopy. (**a**) The relative length/breadth ratio was determined based on the maximum length of the cell divided by the maximum width of the cell as illustrated for uninfected (left panel) or infected cells (right panel). (**b**). The relative length/breadth ratios for each time point were graphed using a Tukey’s box and whisker plot to show the distribution of ratios across time. Ratios that were greater than the 75^th^ percentile +the 1.5 IQR are shown as individual data points. Significance was determined by an Ordinary one-way ANOVA (Supplementary Table S1). Overall, for all time points, AGS cell elongation for all infected strains was significantly increased compared to uninfected or *cagA::cat* infected cells (*P* < 0.0001). At the 3 hr and 6 hr time points, cells infected with strains expressing any number of EPIYA-C motifs or an EPIYA-D motif were significantly increased compared to cells infected with an EPIYA-AB^T^ motif, whereas at the 9 hr time point, only the EPIYA-AB^T^C and WT G27 were significant as compared to the EPIYA-AB^T^ motif (see Supplementary Table S1 for statistical values). (**c**) The relative length/breadth ratios were grouped (0 < 4, 4 < 8, ≥ 8) and the percentage of elongated cells was determined. At 3 hrs post-infection, there was an increase in the percentage of cells that elongated when infected with strains expressing increasing numbers of EPIYA-C motifs or the EPIYA-D motif. However, the percentage of cells that elongated with any EPIYA-C or -D motif plateaued at 6 and 9 hrs post-infection. The data represent three independent experiments.

Following translocation, phosphorylated CagA induces host cell signaling changes that result in a dramatic cell elongation termed the “hummingbird phenotype”^20, 27^. Previous studies suggest that cellular elongation may be effected by variation in the EPIYA region of CagA^46^. To investigate this possibility, AGS cells were infected with the isogenic strains and host cell elongation was monitored temporally by measuring the maximum length and breadth of cells (illustrated in Fig. 2a). Uninfected cells were maintained as controls and remained largely unchanged throughout the experiment with a mean length/breadth ratio of 1.97 to 2.26 (Fig. 2b). Similarly, infection with the *cagA::cat* strain resulted in no significant changes in cell shape (mean length/breadth ratio of 2.43 to 2.66; Fig. 2b and Supplementary Table S1). Conversely, AGS cells infected with strains carrying a functional CagA protein produced elongated cellular projections (Fig. 2a) that resulted in significant time-dependent changes in the length/breadth ratio as compared to uninfected cells or to cells infected with the *cagA::cat* strain (Fig. 2b; Supplementary Table S1). Significant differences in host cell elongation across the strains were most pronounced at 3 hrs post-infection but the degree of elongation appeared more similar at later time points (Supplementary Table S1). In regard to the EPIYA variants, AGS cell elongation was significantly increased at 3 and 6 hrs for all strains expressing CagA that contained at least one EPIYA-C motif or the EPIYA-D motif as compared to cells infected with the EPIYA-AB^T^ strain (Fig. 2b, Supplementary Table S1). Furthermore, at 3 hrs post-infection, strains containing an EPIYA-AB^T^CCC, -AB^T^CCCC, or -AB^T^D motif induced significant changes as compared to EPIYA-AB^T^C infected cells. In this regard, the EPIYA-AB^T^CCCC strain induced more elongation than strains with two EPIYA-C motifs (EPIYA-AB^T^CC and WT G27; Fig. 2, Supplementary Table S1). By 6 hrs the differences between the strains containing at least one EPIYA-C motif or the EPIYA-D motif were no longer apparent. Finally, AGS cells that were infected with the Δ*cagA*, ΔEPIYA, *cagA::cat*, WT G27, or the ABCC Restorant strains showed the expected phenotypes (see Supplementary Fig. S3 and Supplementary Table S2).

As most prior studies that have evaluated EPIYA-dependent changes in AGS cell elongation have focused on the percentage of cells that became elongated upon infection, we also evaluated our data in this manner. For this analysis, we grouped the relative length/breadth ratios based on the length/breadth ratios that were observed for the uninfected control cells at the 3 hr time point. At this point ~95% of uninfected control cells had a length/breadth ratio of less than 4 (Fig. 2c), therefore, all cells with a ratio of less than 4 were grouped together. The remaining cells were grouped such that the degree of elongation could be differentiated as moderate (length/breadth of 4 < 8) or extreme (length/breadth ≥ 8). Based on these criteria, at the 3 hr time point the percentage of moderately elongated cells correlated with an increase in the number of EPIYA-C motifs or the presence of an EPIYA-D motif. However at 6 and 9 hrs post-infection, the percentage of elongated cells plateaued at 60–65% (Fig. 2c). Furthermore, at these later time points, the percentage of cells that had projections between 4 and 8 or greater than 8 appeared similar across the strains with at least one EPIYA-C or an EPIYA-D motif. *En masse*, these results suggest that while variation in the EPIYA-C and EPIYA-D motif affects the timing of induction of host cell elongation, the presence of multiple EPIYA-C motifs or an EPIYA-D motif does not ultimately result in significantly greater levels of host cell elongation in the AGS model.

### Effect of EPIYA variation on IL-8 secretion

**Figure 3:**
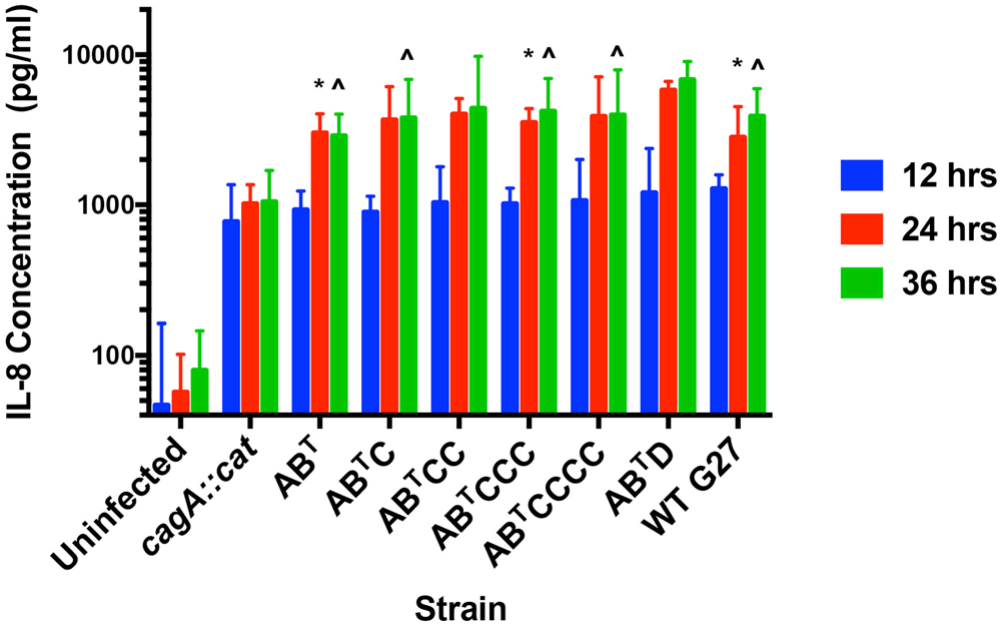
Presence of an EPIYA-D motif induces the greatest IL-8 secretion. AGS cells were infected with the isogenic strains at an MOI of 10. A sample of the co-culture supernatant was taken at 12, 24, and 36 hrs post-infection and analyzed by ELISA for total IL-8. At all time points, there was a significant increase in IL-8 secretion in cells infected with *H. pylori* (*P* = 0.0001 for all comparisons, two-way ANOVA). At 24 and 36 hrs, there was a significant increase in IL-8 secretion from cells infected with *H. pylori* expressing CagA with an intact EPIYA motif as compared to the truncated CagA protein (*P* = 0.0001, two-way ANOVA). There was no difference in secretion from cells infected with increasing EPIYA-C motifs. *Indicates significance compared to EPIYA-AB^T^D at 24 hrs; **^**Indicates significance compared to EPIYA-AB^T^D at 36 hrs. ELISA data are presented as the geometric mean +95% confidence interval and represent three independent experiments.

*H. pylori* infection results in a chronic inflammatory immune response that includes secretion of the cytokine IL-8^47, 48^. While induction of IL-8 is dependent on the presence of the T4SS^8, 49–51^, studies indicate that CagA also contributes to IL-8 secretion at later time points^40, 52–54^. Thus, to determine if CagA EPIYA variation influenced IL-8 secretion in infected AGS cells, IL-8 was monitored at 12, 24, and 36 hrs post-infection (Fig. 3). IL-8 secretion was significantly increased at all time points in cells infected with *H. pylori* as compared to uninfected controls (*P* = 0.0001 for all comparisons). Furthermore, CagA-dependent increases in IL-8 were evident at 24 and 36 hrs post-infection where IL-8 production was significantly higher in isogenic strains expressing an intact EPIYA motif as compared to the *cagA::cat* infected cells (*P* = 0.0001). Strains containing the EPIYA-AB^T^, -AB^T^C, -AB^T^CC, -AB^T^CCC, and -AB^T^CCCC motifs induced virtually identical levels of IL-8 across all time points with no significant differences across these strains and were comparable to WT G27. In contrast, when cells were infected with the EPIYA-AB^T^D strain, IL-8 secretion was significantly higher than with other strains at 24 hrs [EPIYA-AB^T^ (*P* = 0.0026), -AB^T^CCC (*P* = 0.0334), and WT G27 (*P* = 0.0008)], and at 36 hrs [EPIYA-AB^T^ (*P* = 0.0001), EPIYA-AB^T^C (*P* = 0.0088), EPIYA-AB^T^CCC (*P* = 0.0403), EPIYA-AB^T^CCCC (*P* = 0.0179), and WT G27 (*P* = 0.0129)]. As in the previous assays, infection with the ABCC Restorant, *cagA::cat*, ΔEPIYA, and Δ*cagA* strains yielded the expected results (see Supplementary Fig. S4). Together, these results indicate that the CagA EPIYA region is required for maximal IL-8 secretion and suggest that the presence of one or several EPIYA-C motifs does not result in increased IL-8 secretion beyond what is seen in the AB^T^ strain. Conversely, and in contrast to the cell elongation data (Fig. 2), the presence of an EPIYA-D motif results in maximal IL-8 secretion that increases in a temporal fashion.

### Effect of EPIYA Variation on CagA Translocation and Phosphorylation, and on Extracellular Regulated Kinase 1/2 (ERK1/2) Activation

**Figure 4:**
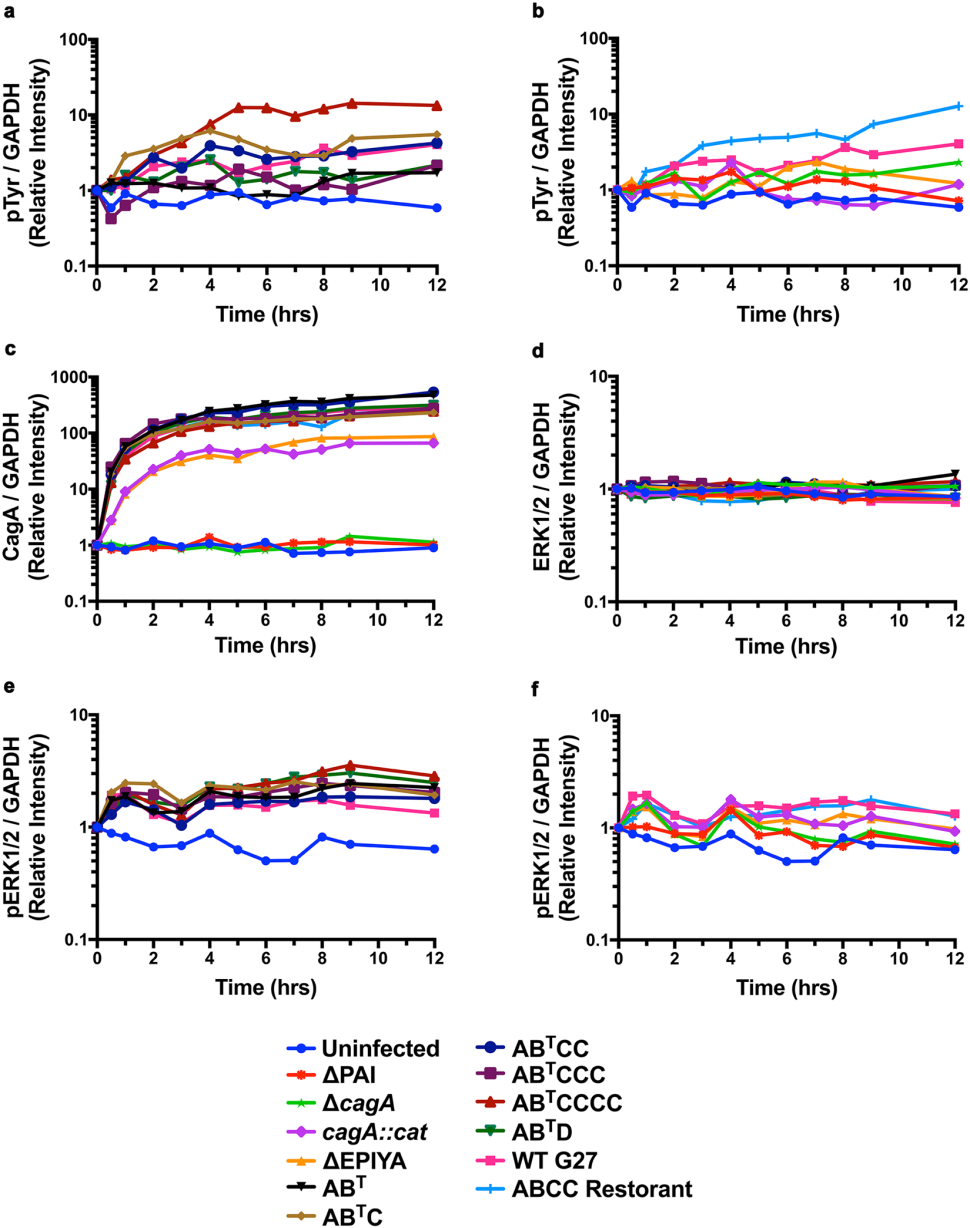
CagA EPIYA polymorphism effects on CagA translocation, phosphorylation, and activation of ERK1/2. AGS cells were infected with the isogenic strains at an MOI of 100 and cells were harvested in a temporal fashion. Lysates were then analyzed for (**a** and **b**) phosphorylated CagA (pTyr), (**c**) total CagA (CagA), (**d**) total ERK1/2 (ERK1/2) and (**e** and **f**) phosphorylated ERK1/2 (pERK1/2). Data were normalized to GAPDH and the value at time zero for each strain for each protein of interest was designated as “1” and variation from that value were then plotted temporally. The data are displayed as the geometric mean of four biologically independent experiments.

Previous studies have shown that CagA that contains multiple EPIYA-C motifs undergoes more tyrosine phosphorylation than variants with a single EPIYA-C motif. Additionally, the presence of multiple EPIYA-C repeats increases binding activity to SHP-2^35, 46^. However, the EPIYA-D motif contains a perfect SHP-2 binding motif and can activate SHP-2 more effectively than an EPIYA-C motif^27, 55, 56^. Therefore, to begin to study how CagA C-terminal polymorphism alters a host cell signaling pathway associated with gastric cancer development, we infected AGS cells with the isogenic strains and utilized Western blot to temporally monitor total CagA, CagA delivery and phosphorylation, and ERK1/2 activation in a temporal manner (see Supplementary Figure S5 for a representative Western blot compilation). As the T4SS is known to activate host cell signaling pathways^57^, we also constructed a G27 ΔPAI strain as an additional comparator. When we quantitated CagA delivery over time, detectable CagA phosphorylation was observed for most strains by 1 hr, increased until the 4–5 hr time point, and then plateaued (Fig. 4a). Higher levels of phosphorylation tended to be observed with the EPIYA-AB^T^CCCC motif followed by the EPIYA-AB^T^CC, -AB^T^C, and WT G27 strains (Fig. 4a). As strains missing CagA (ΔPAI and Δ*cagA*) or containing a truncated CagA (*cagA::cat* and ΔEPIYA) do not contain phosphorylatable residues, the phosphorylation signal clustered with that of the uninfected AGS cells, which was below that of WT G27 and the ABCC Restorant (Fig. 4b). Total CagA increased with time for strains that contained an intact EPIYA motif; polymorphism in the CagA EPIYA motif had no substantial effect on the amount of detectable total CagA between these strains (Fig. 4c). As expected, no CagA signal was detected above background levels for the uninfected controls, the ΔPAI, or the Δ*cagA* infected cells (Fig. 4c). Strains containing a truncated CagA (*cagA::cat* or ΔEPIYA) showed a temporal increase in the truncated form of CagA, however, this increase lagged behind and remained decreased compared to strains with an intact CagA EPIYA region (Fig. 4c). These differences in total CagA between the full length and truncated proteins should be independent of antibody recognition; the polyclonal CagA antibody that was used was raised against amino acids 1–300 of the protein, which are identical in these isogenic strains.

We next monitored ERK1/2 activation by measuring total ERK1/2 and ERK1/2 phosphorylation (pERK1/2). We found that total ERK1/2 remained constant throughout the experiment (Fig. 4d) and that there were no appreciable differences in ERK1/2 activation across strains with an intact EPIYA motif (Fig. 4e). However, we did observe two peaks in ERK1/2 activation in these strains. The first peak occurred 1 hr post-infection and then steadily declined until a second activation peak occurred at 4 hrs post-infection. The later peak remained elevated for all strains with an intact EPIYA region (Fig. 4e and f). The initial peak in ERK1/2 activation can be attributed to the presence of the *cag*-PAI, as the G27 ΔPAI strain did not induce ERK1/2 activation at this time point (Fig. 4f). Together, the data indicate that while polymorphisms in the CagA EPIYA motifs do not appear to affect CagA expression, there is a trend towards increased phosphorylation with strains containing multiple EPIYA-C motifs. Secondly, sustained increases in ERK1/2 activation occurred beginning at 4 hrs post-infection, regardless of EPIYA type. Thus, in this model system CagA-dependent activation of ERK1/2 appears to not be dramatically affected by CagA EPIYA variation.

## Discussion

Based on polymorphism in its C-terminal EPIYA motifs, the oncoprotein CagA is responsible for causing alterations in host cell signaling pathways that impact the development of gastric cancer^17^. However, previous studies have utilized non-isogenic strains or transfection models to study the role of EPIYA polymorphism in infection and gastric cancer development. Recently, a limited panel of isogenic *H. pylori* CagA EPIYA variants was constructed and characterized in the P12 strain background and included EPIYA-AB, -ABC, and -ABCCC motifs, but did not include the more virulent -ABD motif^33, 43, 44^. As such, to begin to determine how EPIYA polymorphism influences gastric cancer development, we created a larger panel of strains in the *H. pylori* G27 strain background; the panel was designed to encompass the most commonly found EPIYA motifs in clinical settings. Additionally, we created control strains that were deficient for CagA or encoded truncated forms of CagA. Characterization of the EPIYA variants showed that CagA was expressed and was translocated into and phosphorylated in AGS cells. Pathway analysis revealed that increasing numbers of EPIYA-C repeats or the presence of the EPIYA-D motif significantly increased host cell elongation, though these differences were only evident in early stages of infection. Furthermore, IL-8 secretion occurred in both a CagA EPIYA-dependent and independent fashion where maximal IL-8 secretion depended on the presence of an EPIYA-ABD motif. We found that levels of CagA phosphorylation trended higher in strains carrying an EPIYA-C motif. Lastly, we found that activation of ERK1/2 occurred in a *cag*-PAI-dependent manner. CagA-independent activation occurred early in infection, while CagA-dependent activation occurred mid-infection and remained sustained regardless of EPIYA type.

The clinical isolate that served as the template for the isogenic strain construction contains a natural alanine to threonine amino acid change in the EPIYA-B motif. At the time of our strain construction, this EPIYA variation was not identified as biologically significant, however a recent publication showed that the EPIYT-B sequence has a higher affinity for the phosphatidylinositol 3-kinase/protein kinase B (PI3K/Akt) pathway and that a strain containing this allele is attenuated for host cell elongation and IL-8 secretion as compared to an isogenic EPIYA-B strain^58^. Zhang *et al*. proposed that the EPIYT-B sequence may allow for increased CagA tyrosine phosphorylation to occur, which in turn, regulates phosphorylation-dependent PI3K/Akt activation to regulate host cell cytoskeletal rearrangements and cytokine secretion^58^. It was also suggested that EPIYT-B motif-induced host cell elongation might relate to a competition in binding between PI3K and C-Src kinase (CSK) or SHP-2 at this domain^58^. We note that while utilization of isogenic strains containing the EPIYT-B motif could impact our results, we did not observe a significant decrease in host cell elongation or IL-8 secretion that appeared linked to the EPIYT polymorphism; data obtained from WT G27 (EPIYA-ABCC), the ABCC Restorant and the isogenic EPIYA-AB^T^CC strain were comparable. Therefore, in the context of our studies, and within the G27 strain background, perhaps any potential increased PI3K/Akt/EPIYT-B binding is over-shadowed by other activated signaling cascades. Further studies will need to be conducted to evaluate this phenomenon. However, our data strongly suggest that our isogenic strain collection can serve as a model system to study CagA EPIYA-mediated changes in host cell signaling pathways.

During *H. pylori* infection, CagA is known to induce the characteristic “hummingbird phenotype” in host epithelial cells^20, 27^. Several studies have proposed that increased levels of CagA phosphorylation correlated with increased host cell elongation^14, 35, 40, 59^. In this regard, transfected CagA constructs with increasing numbers of EPIYA motifs induce greater host cell elongation^35^. Similarly, comparison of non-isogenic strains suggests that levels of host cell elongation are reduced when the number of CagA motifs is reduced^14^. Furthermore, measurement of host cell projections showed that the number of elongated cells correlated with the amount of CagA tyrosine phosphorylation^59^ and that the number and overall length of elongated cells increased with increasing numbers of EPIYA motifs^40^. Lastly, the recent P12 isogenic strain study found that at 24 hrs post-infection, cell scattering was proportional to the number of expressed EPIYA-C motifs^44^. Utilizing our isogenic strain collection and temporal quantitation of host cell elongation, our data suggest that EPIYA-dependent increases in host cell elongation occurs early in infection and that a single EPIYA-C motif is sufficient to initiate maximal host cell elongation at later points. The presence of the CagA EPIYA-D allele does not induce more host cell elongation than the presence of an EPIYA-C allele as the timing and overall host cell elongation patterns were similar among these strains and plateaued identically at the later time points. Thus, utilization of our isogenic strain collection as a means to remove extraneous *H. pylori* variables suggests that within the AGS model system, host cell elongation is influenced by polymorphisms in the CagA EPIYA region only early in infection; however, we do note that it may be possible that lack of a difference in host cell elongation at later time points may be due to saturation of the elongation phenotype in the AGS model. Thus, future analyses in other cell types may be beneficial.

We note that direct comparison of our results to prior studies is difficult due to differences in methodology; despite this, we note the benefit of considering our work within the context of those prior studies. For example, previous data generated using transfected CagA constructs suggest that CagA variants that interact with CSK stronger than with SHP-2 attenuate host cell elongation^56^. That finding suggests that strains that carry the EPIYT-B motifs, which may interact less strongly with the CSK pathway than the isogenic EPIYA-B motif^58^, should result in differences in cell elongation in our assay. However, as previously discussed, we did not observe such a difference. While the reason for this discrepancy is unclear, future work to investigate this will include more extensive pathway characterization. While the significance of the timing of CagA-induced host cell elongation remains unclear, CagA phosphorylation and EPIYA region variations are suggested to result in activation of host cell signaling pathways that contribute to EMT, a hallmark of tumorigenesis that involves the loss of epithelial cell polarization and cell-to-cell contacts and the acquisition of increased motility^34^. CagA’s role in EMT is attributed to inhibition of focal adhesion retraction in the trailing edge of migrating cells^60^, activation of SHP-2, inhibition of PAR1 homolog b (Par1b)^22, 23^, and alteration of EMT markers^32, 33, 61, 62^. In *H. pylori* strains with multiple CagA EPIYA-C motifs, the interaction between CagA and SHP-2 is enhanced^56, 63^, CagA binding to Par1b increases exponentially^64^, and expression and phosphorylation of the EPIYA-C motifs transcriptionally regulates EMT markers and stem cells in a phosphorylation-dependent manner^33^. Thus, it will be interesting to use our constructed isogenic strain panel for future live-cell imaging studies that will investigate EMT specific pathways; additional time points post-infection can be used to more accurately assess host cell elongation kinetics.

Upon infection, *H. pylori* induces host cell inflammation that includes the secretion of cytokines, such as IL-8^47, 48^. IL-8 secretion is induced in response to the T4SS early in infection^8, 49–51^, while a role for CagA has been suggested at later time points^40, 52–54^. However, further work aimed at investigating the role of CagA EPIYA polymorphism in IL-8 secretion has yielded inconsistent results. First, several studies using non-isogenic clinical strains found that EPIYA variation has no significant association with the amount of IL-8 secreted by AGS cells following a short infection (3–6 hrs)^40, 54^. Subsequent studies indicate a role for CagA-dependent, but phosphorylation independent, effects of IL-8 secretion at later time points post-infection (greater than 36 hrs)^53, 65^. Conversely, a study that assessed time points at 6 and 36 hrs post-infection found that increasing numbers of EPIYA-C motifs significantly increased IL-8 secretion and that the increase was even more dramatic in the presence of an EPIYA-ABD motif^38^. Conversely, results from the *H. pylori* P12 isogenic strain collection revealed no difference in IL-8 secretion among strains containing varying numbers of EPIYA-C motifs^44^. Our results utilizing the expanded G27 isogenic strain panel further support the P12 isogenic strain panel findings. We found infection-dependent IL-8 secretion early in infection (12 hrs post-infection) and CagA-dependent IL-8 induction at later time points (>24 hrs post-infection). Regardless of the number of EPIYA-C motifs, the presence of a single EPIYA-C motif was enough to induce maximal IL-8 secretion. However, the amount of IL-8 induced by the EPIYA-ABD strain was the highest. Interestingly, this finding differs from our cell elongation results, where maximal elongation occurred in the presence of either an EPIYA-C or -D motif. *En masse*, our data support the hypothesis that CagA influences IL-8 secretion in an EPIYA-dependent manner where the EPIYA-D motif is critical for maximal induction of secretion. Furthermore, increasing EPIYA-C motifs appear to not greatly influence IL-8 secretion levels in a model of natural infection (refs 43 and 44, and this study).

Upon initial investigation, we observed differences in overall CagA translocation into and phosphorylation in AGS cells. Given that CagA phosphorylation is known to influence changes in host cell signaling that affect EMT, cytokine production, and gastric carcinogenesis, we investigated temporal translocation and phosphorylation of CagA as well as associated ERK1/2 activation. Previous studies have shown that CagA translocation and phosphorylation varies by host cell type; in AGS cells, translocation was observed within 1 hr of infection^66^. A CagA transfection study indicated that overall levels of phosphorylation are proportional to the number of EPIYA-C motifs^56^. Our results are consistent with these observations as phosphorylated CagA was detected for most of the isogenic strains around 1 hr post-infection, temporally increased thereafter, and showed the highest levels in the strain containing the most EPIYA-C motifs. ERK1/2 activation following infection with the various isogenic strains followed a biphasic pattern. The first peak in ERK1/2 activation occurred immediately following infection and was T4SS-dependent, while the second peak occurred several hours post-infection, was sustained throughout the assay, and was CagA-dependent. These results are fairly consistent with previous studies that indicate that CagA has a role in ERK1/2 activation but that this role is most evident later in infection. For example, rapid ERK1/2 activation (within 15 min of infection) was shown to be similar between *H. pylori* strains regardless of CagA status, but CagA positive strains induced sustained levels of ERK1/2 activation^67^. Those authors proposed that CagA tyrosine phosphorylation was required for optimal late ERK1/2 activation and that early CagA-independent activation was mediated by Raf and MEK but not Ras, whereas later activation included all three pathways^67^. Transfected CagA has also been shown to result in prolonged ERK1/2 activation that is mediated by SHP-2^27^. In comparison to our own data, the P12 isogenic strain collection study also found early and late ERK1/2 activation. However, in contrast to our results, early activation appeared to be influenced by the presence of EPIYA-C motifs; the P12 AB strain induced lower levels of early activation as compared to the P12 ABCCC strain. Further, Papadakos *et al*. found that late ERK activation appeared to be highest in the P12 strains lacking EPIYA-C motifs: AB and Δ*cagA* ^43^. Though not completely clear, the reason for the differences between these isogenic strain studies may be based on strain differences (P12 vs G27), experimental setup (RPMI + 10%FBS vs F-12K + 2% FBS), etc. The data presented herein seem to suggest that *H. pylori*-mediated ERK1/2 activation follows a biphasic pattern. Early activation requires the T4SS but not CagA, whereas sustained ERK1/2 activation specifically requires CagA. As previously suggested, and as supported by the overall low levels of CagA phosphorylation we observed in the present study, the CagA toxin appears very potent since it appears to be able to modify host cell signaling pathways with minimal levels of CagA translocation and phosphorylation^63, 68^.

In conclusion, our study supports the paradigm that CagA polymorphisms in the C-terminus affects host cell signaling pathways that vary based on the number of and type of CagA EPIYA-motifs that are expressed. Furthermore, we show that our isogenic strain collection serves as a good model to study differences in host cell signaling pathways of interest. One caveat that must be considered when interpreting our results is that there are additional natural amino acid differences that occur in the C-terminus of CagA that lie outside of the EPIYA motifs (Supplementary Figure S1). Therefore, future studies will be required to determine whether these amino acid differences contribute to CagA-mediated effects as well as to dissect how the EPIYA polymorphisms influence specific pathways that lead to gastric carcinogenesis.

## Methods

### Bacterial strains, plasmids, and eukaryotic cell lines

Strains and plasmids are listed in Table 1. *H. pylori* was cultured as previously described^69^. Briefly, *H. pylori* were grown on blood plates for 24–48 hrs prior to transfer to liquid starter cultures. *H. pylori* liquid starter cultures were grown at 37 °C for 18–24 hrs shaking at 110 rpm in Brucella broth (BB) supplemented with 10% fetal bovine serum (FBS) and 10 µg/mL vancomycin (Vanc). For experimentation, liquid *H. pylori* starter cultures were diluted to 0.05 OD unit/mL (ODU) at 600 nm and cultured for 16–18 hrs. Where denoted in Table 1, cultures and plates were supplemented with 8 µg/mL chloramphenicol (CM), 25 µg/mL kanamycin (Kan), or 5% sucrose (Suc). All *H. pylori* cultures were grown under microaerobic conditions (5% O_2_, 10% CO_2_, and 85% N_2_) established using an Anoxomat gas evacuation and replacement system (Advanced Instruments, Inc., Norwood, MA) in gas evacuation jars. *Escherichia coli* strains were maintained as frozen stocks in Luria Broth (LB) containing 40% glycerol at −80 °C. Plasmids were isolated and purified using Qiagen reagents (Qiagen; Valencia, CA) from cultures grown in the presence of 100 μg/mL ampicillin (Amp), and 25 μg/mL Kan or 8 μg/mL CM.

**Table 1:** Plasmids and strains used in this study.

Plasmid or Strain Designation	Description	Reference
**Plasmids**		
pKSF-II	pEK::*kan-sacB*; Kan^R^	71, 72
pDSM530	pGEMT-Easy::7.13ΔEPIYA; Amp^R^	This study
pDSM531	pGEMT-Easy::7.13ΔEPIYA::*kan-sacB*; Amp^R^, Kan^R^	This study
pDSM532	pGEMT-Easy::AB^T^; Amp^R^	This study
pDSM533	pGEMT-Easy::ABD, Amp^R^	This study
pDSM547	pGEMT-Easy::AB^T^D; Amp^R^	This study
pDSM570	pGEMT-Easy::AB^T^C; Amp^R^	This study
pDSM571	pGEMT-Easy::AB^T^CC; Amp^R^	This study
pDSM572	pGEMT-Easy::AB^T^CCC; Amp^R^	This study
pDSM573	pGEMT-Easy::AB^T^CCCC; Amp^R^	This study
pDSM729	pGEMT-Easy::G27-MA::*cagA::cat*, Amp^R^, Cm^R^	This study
pDSM730	pGEMT-Easy::G27-MA::ΔPAI::*kan*; Amp^R^, Kan^R^	75; This study
pDSM1389	pGEMT-Easy::G27Δ*cagA*; Amp^R^	This study
pDSM1390	pGEMT-Easy::G27Δ*cagA*::*kan-sacB*; Amp^R^, Kan^R^, Suc^S^	This study
pDSM1419	pGEMT-Easy::G27*cagA* EPIYA; Amp^R^	This study
***H. pylori*** **Strains**		
G27	WT *H. pylori*	11, 70
DSM48	7.13; gerbil adapted from the clinical strain B128	73
DSM205	G27-MA *cagA::cat*, Cm^R^	75
DSM206	G27-MA ΔPAI, Kan^R^	75
DSM590	*H. pylori* Korean clinical isolate K3	41
DSM591	*H. pylori* Korean clinical isolate K154	41
DSM713	G27ΔEPIYA::*kan-sacB*; Kan^R^, Suc^S^	This study
DSM714	G27 *cagA*-AB^T^;	This study
DSM715	G27 *cagA*-AB^T^C	This study
DSM716	G27 *cagA*-AB^T^CC	This study
DSM717	G27 *cagA*-AB^T^CCC	This study
DSM718	G27 *cagA*-AB^T^CCCC	This study
DSM719	G27 *cagA*-AB^T^D	This study
DSM721	G27 *cagA::cat;* Cm^R^, results in a truncated CagA product	This study
DSM723	G27 ΔPAI; Kan^R^	This study
DSM1391	G27 Δ*cagA*::*kan-sacB*; Kan^R^, Suc^S^	This study
DSM1420	G27 *cagA*-ABCC Restorant	This study

Amp^R^-ampicillin resistant.

CM^R^-chloramphenicol resistant.

Kan^R^-kanamycin resistant.

Suc^S^-sucrose sensitive.

The AGS (CRL-1739) gastric adenocarcinoma cell line was obtained from the American Type Culture Collection (ATCC; Manassas, Virginia). AGS cells were cultured in Ham’s F-12K Nutrient Mixture, Kaighn’s Mod. with L-glutamine (F-12K; Corning Cellgro, Mediatech, Inc., Manassas, VA) supplemented with 10% FBS and 10 µg/mL Vanc at 37 °C, 5% CO_2_ as per ATCC recommendations. For all experiments, AGS cells were seeded in 6-well tissue culture plates.

### *H. pylori* Isogenic Strain Construction

All *H. pylori* strains were constructed in the G27 strain background^11, 70^ using splicing by overlap extension (SOE) PCR with the primers listed in Supplementary Table S3. Detailed methods for the isogenic strain construction can be found in the Supplementary Methods online. Briefly, the C-terminal EPIYA region of WT G27 was replaced with a counter-selectable kanamycin resistance determinant *aphA3/sacB* (*kan*-*sacB*)^71, 72^ to create G27 ΔEPIYA. The C-terminal EPIYA region and motifs were individually PCR amplified from clinical isolates 7.13, K154, and K3^41, 73^. Strain 7.13, a gerbil adapted isolate that causes cancer in the gerbil model of infection^73^, was utilized as the template for the upstream and downstream EPIYA flanking regions to facilitate future creation of *H. pylori* strains that colonize animals. Korean clinical isolates K154 and K3 contain the templates used for the EPIYA-A, -B, and -C or EPIYA-ABD motifs, respectively^41^. The constructs were transformed into G27 ΔEPIYA and transformants were selected for by growth on sucrose; the *kan*-*sacB* cassette was replaced by double homologous recombination of the various PCR amplified EPIYA regions. Subsequently, *cagA* was amplified from each of the constructed strains and was Sanger sequenced. These sequences were then translated and the resulting amino acid sequences were analyzed using Seaview (V.4) to verify that changes in CagA occurred in the C-terminal EPIYA region. A CagA alignment from each of the isogenic strains is provided (Supplementary Figure S1). As controls, a strain in which *cagA* is deleted (Δ*cagA*), a strain where the *cag*-pathogenicity island is deleted (ΔPAI), a strain where *cagA* was disrupted with a chloramphenicol acetyltransferase (*cat*) cassette (*cagA::cat*), and a strain that was restored to the original G27 EPIYA genotype (ABCC Restorant) were also constructed.

### Isogenic Strain CagA Expression, Translocation, and Phosphorylation

To assess CagA expression by the isogenic strains, an aliquot of each liquid culture was pelleted and resuspended in lysis buffer (150 mM NaCl, 50 mM Tris-HCl, pH 8.0, 5 mM EDTA, 1% SDS, 10% glycerol, and Mini-Complete EDTA-Free Proteinase Inhibitor Cocktail). Equal amounts of protein, as determined by a Pierce BCA protein assay, were electrophoresed on 4–12% or 8% Bis-Tris gels (Novex by Life Technologies; Grand Island, NY) and transferred to nitrocellulose membranes using the iBlot Gel Transfer Device (Novex by Life Technologies); membranes were blocked with phosphate buffered saline (PBS) containing 0.05% tween (PBST) and 5% bovine serum albumin Fraction V (BSA). The membranes were probed with a 1:2,000 dilution of rabbit polyclonal IgG anti-CagA (b-300) (Cat #SC-25766, Lot G2413; Santa Cruz Biotechnology Inc., Dallas, TX) and detected with a 1:8,000 dilution of a bovine anti-rabbit IgG-HRP secondary (mouse/human absorbed; Cat #SC2374, lot #I0710; Santa Cruz Biotechnology Inc.).

CagA translocation and phosphorylation by the isogenic strains was evaluated by infection of AGS cells seeded (3.0–3.5 × 10^5^) 36–48 hrs prior to infection. Briefly, the AGS cells were washed twice with PBS and the culture medium was replaced with F-12K (Mediatech Inc.) containing 10% BB, 10% FBS, and 10 µg/mL Vanc, and infected at a multiplicity of infection (MOI) of 100 for 8 hrs. Following co-culture, the medium was removed, the cells were washed twice with PBS to remove non-adherent *H. pylori*, and cells were lysed (150 mM NaCl, 50 mM Tris-HCl, pH 8.0, 5 mM EDTA, 1% SDS, 10% glycerol, Mini-Complete EDTA-Free Proteinase Inhibitor Cocktail, and 0.1 mM Na_3_VO_4_). To detect total and phosphorylated CagA, lysates of equal protein concentration were electrophoresed on 6% SDS gels and then transferred to nitrocellulose membranes. Membranes were blocked with PBST/5% BSA. Phosphorylated CagA was detected using pTyr (P-Tyr-100) mouse mAB (Cat #9411 S; lot #25; Cell Signaling Technology, Danvers, MA) and goat anti-mouse IgG-HRP secondary (Cat #SC-2055; Lot #F2413; Santa Cruz Biotechnology Inc.). Following detection of phosphorylated CagA, the membranes were stripped (2% SDS, 62.5 mM Tris-HCl, pH 6.8, 10 mM DTT) and reprobed for total CagA as described above. All antibodies were detected with the Pierce ECL Western Blotting Substrate on an ImageQuant LAS4000 Luminescent Image Analyzer (GE Healthcare; Laurel, MD) using ImageQuant LAS4000 Software (GE Healthcare).

### Host Cell Elongation

AGS cells were seeded (2.2–2.5 × 10^5^) 18–24 hrs prior to co-culture on collagen-coated coverslips and prepared for infection and inoculated as described above. Three, 6, and 9 hrs post-infection, the coverslips were washed twice with PBS to remove non-adherent bacteria, fixed for 10 min (2.5% paraformaldehyde pH 7.4, 60.75 mM Na_2_HPO_4_, pH 9, and 14.3 mM NaH_2_PO_4_, pH 4.1), washed with PBS, and mounted using Vectashield (Vector Laboratories, Inc., Burlingame, CA). Twenty-four random fields per strain were imaged at 63X using differential interference contrast (DIC) microscopy on a Zeiss LSM 5 Pascal (Carl Zeiss Microscopy, LLC.; Thornwood, NY) or a Leica AF6000 (Leica Microsystems, Buffalo Grove, IL). To determine the relative length/breadth ratio, a total of 200–715 cells/strain were measured from three to six independent experiments. For each cell, maximal length was measured along the longest cellular projection and maximal width was measured at the widest portion of the cell (adapted from^60^) using the LSM 5 Image Browser software (Carl Zeiss Microscopy, LLC.) or the Leica LAS X software (Leica Microsystems, Inc.). Images were exported as TIFF files into Adobe Photoshop CC (Adobe Systems Inc., San Jose, CA) where they were cropped and adjusted using the levels function for contrast and brightness. Data and images represent three to six independent experiments.

### IL-8 Secretion

AGS cells were seeded at 4.2 × 10^5^ for 48 hours. To eliminate activation of host cell signaling pathways due to serum components, the cells were washed twice with PBS and serum starved in F-12K (Mediatech, Inc.) for 2 hrs prior to infection. Our preliminary studies also indicated that an FBS concentration of 10% masked activation of IL-8 by CagA. Therefore, for infection, the serum free medium was replaced with F-12K (Mediatech, Inc.) supplemented with 2% FBS, 10% BB, and 10 µg/mL Vanc, and AGS cells were infected at an MOI of 10. A sample of the co-culture supernatant was harvested every 12 hrs for 36 hrs, centrifuged at 14,000 rpm to pellet any floating cells, and frozen at −20 °C until analysis. The co-culture supernatants were analyzed using the Human CXCL8/IL-8 Quantikine ELISA kit (R&D Systems; Minneapolis, MN) as per manufacturer’s instructions to determine levels of IL-8. ELISA plates were read at an absorbance of 450 nm on a SpectraMax M2 plate reader (Molecular Devices; Downingtown, PA). Results represent three to six independent experiments. Of note, to control for lot specific differences in absolute values of IL-8 identified with the ELISA kit, we included WT G27 in each of our sets of experiments (EPIYA variants in one set and controls in another set) so that we could then compare across the experiments.

### CagA Translocation, Phosphorylation, and Induction of ERK1/2 Activation

AGS cells were seeded (3.0 × 10^5^) for 36–48 hrs. Our preliminary studies indicated that changing the medium on the cells prior to infection activated ERK1/2 and that an FBS concentration of 10% masked activation of ERK1/2 by CagA. Therefore, to determine baseline ERK1/2 activation, a set of AGS cells was harvested prior to changing the media as described below. Next, AGS cells were washed twice with PBS and the medium replaced with F-12K (Mediatech, Inc.) supplemented with 2% FBS, 10% BB, and 10 µg/mL Vanc and a set of samples harvested again to determine activation of ERK1/2 due to serum components. Following the medium change, the cells were then incubated for 3 hrs to allow ERK1/2 activation to return to baseline. Immediately prior to infection, a third set of samples was taken for baseline comparisons (time 0) for the time course. AGS cells were then infected at an MOI of 100 and samples were harvested temporally. At each time point, the medium was removed, the cells washed twice with PBS, and lysed as described above. During harvesting, the lysates were placed on ice followed by sonication in an ice-water bath and frozen at −20 °C until analysis. Samples were electrophoresed on 8% Bis-Tris gels (Novex by Life Technologies) and transferred to nitrocellulose membranes as described. Membranes were blocked with Tris-buffered saline (TBS) containing 0.05% tween (TBST) and 5% BSA, and cut in half horizontally between the 49 and 62 kDa ladders. The upper membranes were probed with a 1:500 dilution of pTyr (PY99) mouse mAB (Cat #SC-7020 lot #D0115 or C2416; Santa Cruz Biotechnology Inc.) and goat anti-mouse IgG-HRP secondary as above. The membranes were then stripped with Re-Blot Plus Strong Solution (Millipore, Billerica, MA), prior to detection of total CagA as previously described. The lower membranes were probed for phosphorylated ERK1/2 (pERK1/2), which was detected using a 1:1,000 dilution of P-p44/42 MAPK (T202/Y204) (D.13.14.4E) XP(R) rabbit mAB (Cat #4370 S; lots #15 and 17; Cell Signaling Technology). The membranes were stripped with Re-Blot Plus Strong Solution (Millipore), prior to detection of total ERK1/2 using 1:1,000 dilution of p44/42 MAPK (ERK1/2) (137F5) rabbit mAB (Cat #4695 S; lot #21; Cell Signaling Technology). Both P-p44/42 MAPK and p44/42 MAPK were detected with a 1:20,000 goat anti-rabbit IgG-HRP (Cat #SC-2004; Lot #F2215; Santa Cruz Biotechnology, Inc.). GAPDH was utilized as a protein loading control and was detected with goat polyclonal anti-GAPDH IgG (L-20; Cat #SC-31915; Lots #D1114 and C1808; Santa Cruz Biotechnology, Inc.) and donkey anti-goat IgG-HRP (Cat #SC-2020; Lot #G1615; Santa Cruz Biotechnology, Inc.). Antibodies were detected with Hy-Glo Quick Spray (Denville Scientific) as previously described. Due to experimental variation across the four independent data sets, the data are displayed as the geometric mean of four biologically independent experiments and are discussed as trends.

### Data analysis and statistics

GraphPad Prism 7 (GraphPad Software, Inc., La Jolla, CA) was used for all graphs and statistical analysis. To identify differences in host cell elongation, the individual length/breadth ratios were grouped by infecting strain or condition, log transformed, and analyzed using an Ordinary one-way analysis of variance (ANOVA; GraphPad Software, Inc.). A Tukey’s multiple comparison test with a single pooled variance was used to assess multiple comparisons (GraphPad Software, Inc.). The data are graphed as a Tukey’s box and whisker plot where any value greater than the 75^th^ percentile plus the 1.5 interquartile distance (IQR) is represented as an individual point. To determine the percentage of cells that were elongated under each condition, the individual length/breadth ratios were grouped as 0 < 4, 4 < 8, and ≥8. These groupings were selected based on analysis of the relative length/breadth ratios of the uninfected control cells at the 3 hr time point so that only ~5% of the uninfected cells were elongated or had a length/breadth ratio of ≥4. The number of cells in each grouping were then divided by the total number of ratios to determine percentages and were graphed as a stacked bar graph. Differences in IL-8 ELISA antibody titers were determined on log transformed data by a two-way ANOVA with repeated measures for time (GraphPad Software, Inc.). To test the hypothesis that differences exist in IL-8 secretion between the EPIYA-AB^T^ and EPIYA-C alleles, multiple comparisons were evaluated with a Tukey’s multiple comparison test (GraphPad Software, Inc.). To test the hypothesis that IL-8 secretion is increased due to the presence of the EPIYA-D allele, multiple comparisons were corrected for using Dunnett’s test (GraphPad Software, Inc.). Dunnett’s test was also used to compare IL-8 secretion for all infected cells to uninfected cells and IL-8 secretion from cells infected with strains containing an intact EPIYA motif to the *cagA::cat* infected cells. Multiple comparisons among control strains were tested using the Tukey’s multiple comparison test (GraphPad Software, Inc.). ELISA data are displayed as the geometric mean +95% confidence interval^74^. Western blot signals were quantified using ImageJ version 1.50 g (National Institutes of Health). Background was removed using the subtract background command (rolling ball correction method with a radius set to 25 pixels) and the signal from the individual bands was measured using the mean from a region of interest drawn around the band. The box size for measuring was the same for the phosphorylated versus total form of the protein. Data for each protein of interest were normalized to GAPDH. Due to variation between strains and biological replicates for each protein of interest, the normalized value at time zero was designated as “1” and then data were plotted temporally as the geometric mean.

### Data availability

The datasets generated during and/or analyzed during the current study are available from the corresponding author on reasonable request.

## Electronic supplementary material

Supplementary Material
