# Supplementary material for: Creation and Initial Characterization of Isogenic Helicobacter pylori CagA EPIYA Variants Reveals Differential Activation of Host Cell Signaling Pathways
Source: Sci Rep. 2017 Sep 8;7:11057. doi: 10.1038/s41598-017-11382-y (PMC5591203; doi:10.1038/s41598-017-11382-y)
Supplement: Supplementary file 1 — Supplementary Material [file 41598_2017_11382_MOESM1_ESM.pdf]

Creation and Initial Characterization of Isogenic *Helicobacter pylori* CagA EPIYA Variants Reveals Differential Activation of Host Cell Signaling Pathways

Dacie R. Bridge[1,+], Faith C. Blum[1], Sungil Jang[2], Jinmoon Kim[2,3], Jeong-Heon Cha[2,3,4], and D. Scott Merrell[1*]

Supplementary Results and Methods

SUPPLEMENTARY RESULTS

1

AB^{T}	MTNETINQQP	QTEAAFNPQQ	FINNLQVAFL	KVDNAVASYD	PDQKPIVDKN	DRDNRQAFNG	ISQLREEYSN
$AB^{T}C$	MTNETINQQP	QTEAAFNPQQ	FINNLQVAFL	KVDNAVASYD	PDQKPIVDKN	DRDNRQAFNG	ISQLREEYSN
$AB^{T}CC$	MTNETINQQP	QTEAAFNPQQ	FINNLQVAFL	KVDNAVASYD	PDQKPIVDKN	DRDNRQAFNG	ISQLREEYSN
$AB^{T}CCC$	MTNETINQQP	QTEAAFNPQQ	FINNLQVAFL	KVDNAVASYD	PDQKPIVDKN	DRDNRQAFNG	ISQLREEYSN
$AB^{T}CCCC$	MTNETINQQP	QTEAAFNPQQ	FINNLQVAFL	KVDNAVASYD	PDQKPIVDKN	DRDNRQAFNG	ISQLREEYSN
$AB^{T}D$	MTNETINQQP	QTEAAFNPQQ	FINNLQVAFL	KVDNAVASYD	PDQKPIVDKN	DRDNRQAFNG	ISQLREEYSN
WT G27	MTNETINQQP	QTEAAFNPQQ	FINNLQVAFL	KVDNAVASYD	PDQKPIVDKN	DRDNRQAFNG	ISQLREEYSN
7.13	MTNETINQQP	QTEAAFNPQQ	FINNLQVAFI	KVDNAVASFD	PDQKPIVDKN	DRDNRQAFEK	ISQLREKFAN

71

AB^{T}	KAIKNPAKKN	QYFSDFIDKS	NNLINKDALI	DVESSTKSFQ	KFGDQRYQIF	TSWVSHQNDP	SKINTRSIRN
$AB^{T}C$	KAIKNPAKKN	QYFSDFIDKS	NNLINKDALI	DVESSTKSFQ	KFGDQRYQIF	TSWVSHQNDP	SKINTRSIRN
$AB^{T}CC$	KAIKNPAKKN	QYFSDFIDKS	NNLINKDALI	DVESSTKSFQ	KFGDQRYQIF	TSWVSHQNDP	SKINTRSIRN
$AB^{T}CCC$	KAIKNPAKKN	QYFSDFIDKS	NNLINKDALI	DVESSTKSFQ	KFGDQRYQIF	TSWVSHQNDP	SKINTRSIRN
$AB^{T}CCCC$	KAIKNPAKKN	QYFSDFIDKS	NNLINKDALI	DVESSTKSFQ	KFGDQRYQIF	TSWVSHQNDP	SKINTRSIRN
$AB^{T}D$	KAIKNPAKKN	QYFSDFIDKS	NNLINKDALI	DVESSTKSFQ	KFGDQRYQIF	TSWVSHQNDP	SKINTRSIRN
WT G27	KAIKNPAKKN	QYFSDFIDKS	NNLINKDALI	DVESSTKSFQ	KFGDQRYQIF	TSWVSHQNDP	SKINTRSIRN
7.13	KAIKNPTKKN	QYFSNFISKS	SDLINKDGLI	DTGSSIKSFQ	KFGTQRYQIF	MNWVSHQKDP	SQINTQKIRG

141

AB^{T}	FMENIIQPPI	PDDKEKAEFL	KSAKQSFAGI	IIGNQIRTDQ	KFMGVFDESL	KERQEAEKNG	G----PTGGD
$AB^{T}C$	FMENIIQPPI	PDDKEKAEFL	KSAKQSFAGI	IIGNQIRTDQ	KFMGVFDESL	KERQEAEKNG	G----PTGGD
$AB^{T}CC$	FMENIIQPPI	PDDKEKAEFL	KSAKQSFAGI	IIGNQIRTDQ	KFMGVFDESL	KERQEAEKNG	G----PTGGD
$AB^{T}CCC$	FMENIIQPPI	PDDKEKAEFL	KSAKQSFAGI	IIGNQIRTDQ	KFMGVFDESL	KERQEAEKNG	G----PTGGD
$AB^{T}CCCC$	FMENIIQPPI	PDDKEKAEFL	KSAKQSFAGI	IIGNQIRTDQ	KFMGVFDESL	KERQEAEKNG	G----PTGGD
$AB^{T}D$	FMENIIQPPI	PDDKEKAEFL	KSAKQSFAGI	IIGNQIRTDQ	KFMGVFDESL	KERQEAEKNG	G----PTGGD
WT G27	FMENIIQPPI	PDDKEKAEFL	KSAKQSFAGI	IIGNQIRTDQ	KFMGVFDESL	KERQEAEKNG	G----PTGGD
7.13	FMENIIQPPI	SDDKEKAEFL	RSAKQALAGI	IIRNQIRSDQ	KFMGVFDESL	KERQEAEKNG	EPNGDPTGGD

211

AB^{T}	WLDIFLSFIF	DKKQSSDVKE	AINQEPVPHV	QPDIATTTTD	IQGLPPEARD	LLDERGNFSK	FTLGDMEMLD
$AB^{T}C$	WLDIFLSFIF	DKKQSSDVKE	AINQEPVPHV	QPDIATTTTD	IQGLPPEARD	LLDERGNFSK	FTLGDMEMLD
$AB^{T}CC$	WLDIFLSFIF	DKKQSSDVKE	AINQEPVPHV	QPDIATTTTD	IQGLPPEARD	LLDERGNFSK	FTLGDMEMLD
$AB^{T}CCC$	WLDIFLSFIF	DKKQSSDVKE	AINQEPVPHV	QPDIATTTTD	IQGLPPEARD	LLDERGNFSK	FTLGDMEMLD
$AB^{T}CCCC$	WLDIFLSFIF	DKKQSSDVKE	AINQEPVPHV	QPDIATTTTD	IQGLPPEARD	LLDERGNFSK	FTLGDMEMLD
$AB^{T}D$	WLDIFLSFIF	DKKQSSDVKE	AINQEPVPHV	QPDIATTTTD	IQGLPPEARD	LLDERGNFSK	FTLGDMEMLD
WT G27	WLDIFLSFIF	DKKQSSDVKE	AINQEPVPHV	QPDIATTTTD	IQGLPPEARD	LLDERGNFSK	FTLGDMEMLD
7.13	WFDIFLSFVF	NKKQSSDLKE	TLNQEPVPHV	QPDVATTTTD	IQSLPPESRD	LLDERGNFSK	FTLGDMEMLD

281

AB^{T}	VEGVADIDPN	YKFNQLLIHN	NALSSVLMGS	HNGIEPEKVS	LLYGGNGGPK	AKHDWNATVG	YKDQQGNNVA
$AB^{T}C$	VEGVADIDPN	YKFNQLLIHN	NALSSVLMGS	HNGIEPEKVS	LLYGGNGGPK	AKHDWNATVG	YKDQQGNNVA
$AB^{T}CC$	VEGVADIDPN	YKFNQLLIHN	NALSSVLMGS	HNGIEPEKVS	LLYGGNGGPK	AKHDWNATVG	YKDQQGNNVA
$AB^{T}CCC$	VEGVADIDPN	YKFNQLLIHN	NALSSVLMGS	HNGIEPEKVS	LLYGGNGGPK	AKHDWNATVG	YKDQQGNNVA
$AB^{T}CCCC$	VEGVADIDPN	YKFNQLLIHN	NALSSVLMGS	HNGIEPEKVS	LLYGGNGGPK	AKHDWNATVG	YKDQQGNNVA
$AB^{T}D$	VEGVADIDPN	YKFNQLLIHN	NALSSVLMGS	HNGIEPEKVS	LLYGGNGGPK	AKHDWNATVG	YKDQQGNNVA
WT G27	VEGVADIDPN	YKFNQLLIHN	NALSSVLMGS	HNGIEPEKVS	LLYGGNGGPK	AKHDWNATVG	YKDQQGNNVA
7.13	VEGVADIDPN	YKFNQLLVHN	NALSSVLMGS	HDGIEPEKVS	LLYGNNGGPE	ARHDWNATVG	HKNQQGNNVA

351

AB^{T}	TIINVHMKNG	SGLVIAGGEK	GINNPSFYLY	KEDQLTGSQR	ALSQEEIRNK	VDFMEFLAQN	NAKLDNLSEK
$AB^{T}C$	TIINVHMKNG	SGLVIAGGEK	GINNPSFYLY	KEDQLTGSQR	ALSQEEIRNK	VDFMEFLAQN	NAKLDNLSEK
$AB^{T}CC$	TIINVHMKNG	SGLVIAGGEK	GINNPSFYLY	KEDQLTGSQR	ALSQEEIRNK	VDFMEFLAQN	NAKLDNLSEK
$AB^{T}CCC$	TIINVHMKNG	SGLVIAGGEK	GINNPSFYLY	KEDQLTGSQR	ALSQEEIRNK	VDFMEFLAQN	NAKLDNLSEK
$AB^{T}CCCC$	TIINVHMKNG	SGLVIAGGEK	GINNPSFYLY	KEDQLTGSQR	ALSQEEIRNK	VDFMEFLAQN	NAKLDNLSEK
$AB^{T}D$	TIINVHMKNG	SGLVIAGGEK	GINNPSFYLY	KEDQLTGSQR	ALSQEEIRNK	VDFMEFLAQN	NAKLDNLSEK
WT G27	TIINVHMKNG	SGLVIAGGEK	GINNPSFYLY	KEDQLTGSQR	ALSQEEIRNK	VDFMEFLAQN	NAKLDNLSEK
7.13	TLINVHMKNG	SGLVIAGGEK	GVNNPSFYLY	KEDQLTGLKQ	ALSQKEIQNK	VDFMEFLAQN	NAKLDNLSKK

421

AB^{T}	EEEKFRNEIK	DFQKDSKAYL	DALGNDRIAF	VSKKDTKHSA	LITEFGNGDL	SYTLKDYGKK	ADKALDREKN
$AB^{T}C$	EEEKFRNEIK	DFQKDSKAYL	DALGNDRIAF	VSKKDTKHSA	LITEFGNGDL	SYTLKDYGKK	ADKALDREKN
$AB^{T}CC$	EEEKFRNEIK	DFQKDSKAYL	DALGNDRIAF	VSKKDTKHSA	LITEFGNGDL	SYTLKDYGKK	ADKALDREKN
$AB^{T}CCC$	EEEKFRNEIK	DFQKDSKAYL	DALGNDRIAF	VSKKDTKHSA	LITEFGNGDL	SYTLKDYGKK	ADKALDREKN
$AB^{T}CCCC$	EEEKFRNEIK	DFQKDSKAYL	DALGNDRIAF	VSKKDTKHSA	LITEFGNGDL	SYTLKDYGKK	ADKALDREKN
$AB^{T}D$	EEEKFRNEIK	DFQKDSKAYL	DALGNDRIAF	VSKKDTKHSA	LITEFGNGDL	SYTLKDYGKK	ADKALDREKN
WT G27	EEEKFRNEIK	DFQKDSKAYL	DALGNDRIAF	VSKKDTKHSA	LITEFGNGDL	SYTLKDYGKK	ADKALDREKN
7.13	EKEKFQNEIE	DFQKDHKAYL	DALGNDHIAF	VSKKDKKHLA	LVTEFGSGDL	SYTLKDYGKK	ADKALDREIK

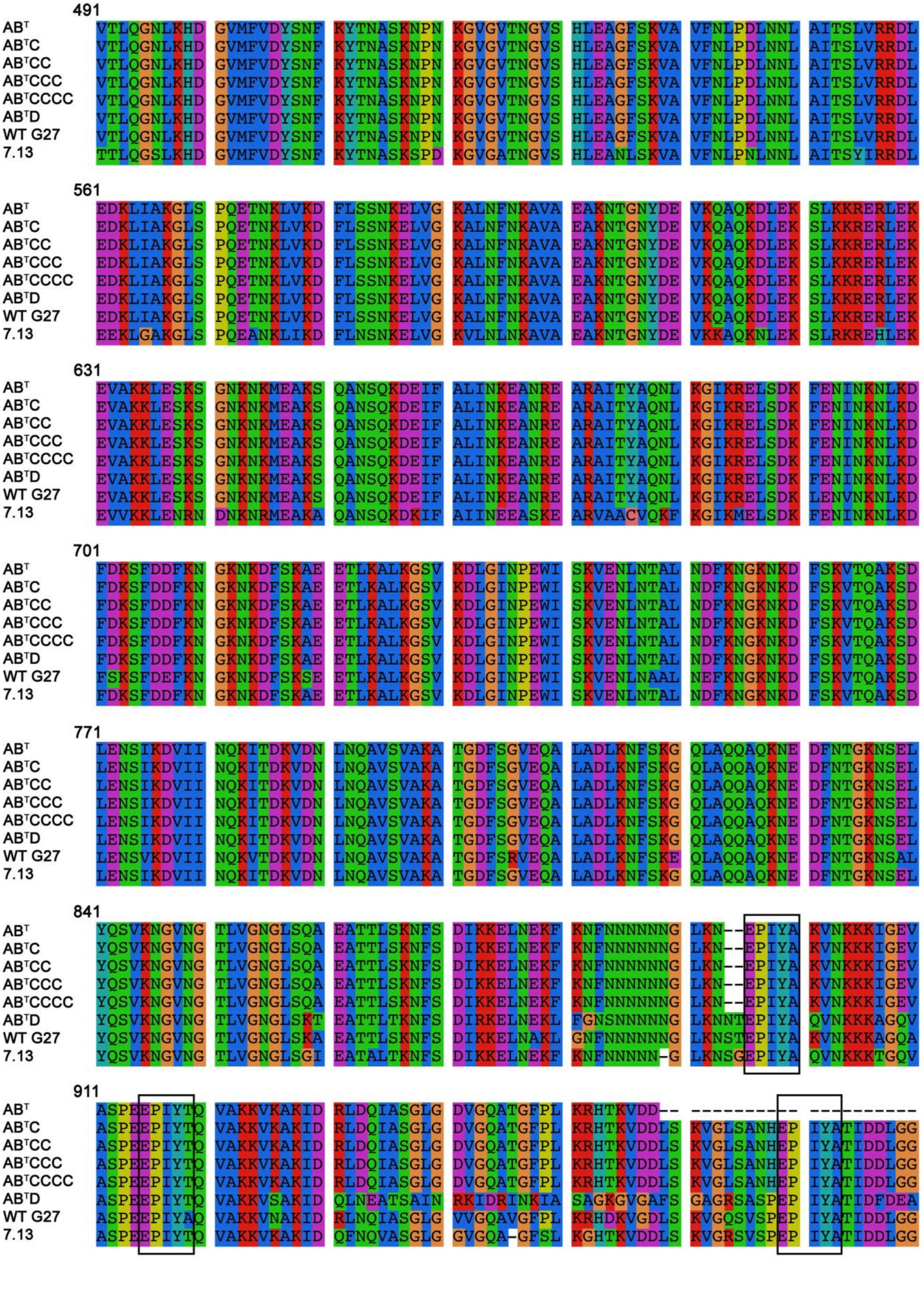
491
AB^{T} VTLQGNLKHD GVMFVDYSNF KYTNASKNPN KGVGVTNGVS HLEAGFSKVA VFNLPDLNNL AITSLVRRDL
$AB^{T}C$ VTLQGNLKHD GVMFVDYSNF KYTNASKNPN KGVGVTNGVS HLEAGFSKVA VFNLPDLNNL AITSLVRRDL
$AB^{T}CC$ VTLQGNLKHD GVMFVDYSNF KYTNASKNPN KGVGVTNGVS HLEAGFSKVA VFNLPDLNNL AITSLVRRDL
$AB^{T}CCC$ VTLQGNLKHD GVMFVDYSNF KYTNASKNPN KGVGVTNGVS HLEAGFSKVA VFNLPDLNNL AITSLVRRDL
$AB^{T}CCCC$ VTLQGNLKHD GVMFVDYSNF KYTNASKNPN KGVGVTNGVS HLEAGFSKVA VFNLPDLNNL AITSLVRRDL
$AB^{T}D$ VTLQGNLKHD GVMFVDYSNF KYTNASKNPN KGVGVTNGVS HLEAGFSKVA VFNLPDLNNL AITSLVRRDL
WT G27 VTLQGNLKHD GVMFVDYSNF KYTNASKNPN KGVGVTNGVS HLEAGFSKVA VFNLPDLNNL AITSLVRRDL
7.13 TTLQGSLKHD GVMFVDYSNF KYTNASKSPD KGVGATNGVS HLEANLSKVA VFNLPNLNNL AITSYIRRDL
561
AB^{T} EDKLIAKGLS PQETNKLVKD FLSSNKELVG KALNFNKAVA EAKNTGNYDE VKQAQKDLEK SLKKRERLEK
$AB^{T}C$ EDKLIAKGLS PQETNKLVKD FLSSNKELVG KALNFNKAVA EAKNTGNYDE VKQAQKDLEK SLKKRERLEK
$AB^{T}CC$ EDKLIAKGLS PQETNKLVKD FLSSNKELVG KALNFNKAVA EAKNTGNYDE VKQAQKDLEK SLKKRERLEK
$AB^{T}CCC$ EDKLIAKGLS PQETNKLVKD FLSSNKELVG KALNFNKAVA EAKNTGNYDE VKQAQKDLEK SLKKRERLEK
$AB^{T}CCCC$ EDKLIAKGLS PQETNKLVKD FLSSNKELVG KALNFNKAVA EAKNTGNYDE VKQAQKDLEK SLKKRERLEK
$AB^{T}D$ EDKLIAKGLS PQETNKLVKD FLSSNKELVG KALNFNKAVA EAKNTGNYDE VKQAQKDLEK SLKKRERLEK
WT G27 EDKLIAKGLS PQETNKLVKD FLSSNKELVG KALNFNKAVA EAKNTGNYDE VKQAQKDLEK SLKKRERLEK
7.13 EEKLGAKGLS PQEANKLIKD FLNSNKELVG KVLNLNKAVA EAKNTGNYDE VKKAQKNLEK SLRKREHLEK
631
AB^{T} EVAKKLESKS GNKNKMEAKS QANSQKDEIF ALINKEANRE ARAITYAQNL KGIKRELSDK FENINKNLKD
$AB^{T}C$ EVAKKLESKS GNKNKMEAKS QANSQKDEIF ALINKEANRE ARAITYAQNL KGIKRELSDK FENINKNLKD
$AB^{T}CC$ EVAKKLESKS GNKNKMEAKS QANSQKDEIF ALINKEANRE ARAITYAQNL KGIKRELSDK FENINKNLKD
$AB^{T}CCC$ EVAKKLESKS GNKNKMEAKS QANSQKDEIF ALINKEANRE ARAITYAQNL KGIKRELSDK FENINKNLKD
$AB^{T}CCCC$ EVAKKLESKS GNKNKMEAKS QANSQKDEIF ALINKEANRE ARAITYAQNL KGIKRELSDK FENINKNLKD
$AB^{T}D$ EVAKKLESKS GNKNKMEAKS QANSQKDEIF ALINKEANRE ARAITYAQNL KGIKRELSDK FENINKNLKD
WT G27 EVAKKLESKS GNKNKMEAKS QANSQKDEIF ALINKEANRE ARAITYAQNL KGIKRELSDK LENVNKNLKD
7.13 EVVKKLENRN DNKNRMEAKA QANSQKDKIF AIINEEASKE ARVAACVQKF KGIKMELSDK FENINKNLKD
701
AB^{T} FDKSFDDFKN GKNKDFSKAE ETLKALKGSV KDLGINPEWI SKVENLNTAL NDFKNGKNKD FSKVTQAKSD
$AB^{T}C$ FDKSFDDFKN GKNKDFSKAE ETLKALKGSV KDLGINPEWI SKVENLNTAL NDFKNGKNKD FSKVTQAKSD
$AB^{T}CC$ FDKSFDDFKN GKNKDFSKAE ETLKALKGSV KDLGINPEWI SKVENLNTAL NDFKNGKNKD FSKVTQAKSD
$AB^{T}CCC$ FDKSFDDFKN GKNKDFSKAE ETLKALKGSV KDLGINPEWI SKVENLNTAL NDFKNGKNKD FSKVTQAKSD
$AB^{T}CCCC$ FDKSFDDFKN GKNKDFSKAE ETLKALKGSV KDLGINPEWI SKVENLNTAL NDFKNGKNKD FSKVTQAKSD
$AB^{T}D$ FDKSFDDFKN GKNKDFSKAE ETLKALKGSV KDLGINPEWI SKVENLNTAL NDFKNGKNKD FSKVTQAKSD
WT G27 FSKSFDEFKN GKNKDFSKSE ETLKALKGSV KDLGINPEWI SKVENLNAAL NEFKNGKNKD FSKVTQAKSD
7.13 FDKSFDDFKN GKNKDFSKAE ETLKALKGSV KDLGINPEWI SKVENLNTAL NDFKNGKNKD FSKVTQAKSD
771
AB^{T} LENSIKDVII NQKITDKVDN LNQAVSVAKA TGDFSGVEQA LADLKNFSKG QLAQQAQKNE DFNTGKNSEL
$AB^{T}C$ LENSIKDVII NQKITDKVDN LNQAVSVAKA TGDFSGVEQA LADLKNFSKG QLAQQAQKNE DFNTGKNSEL
$AB^{T}CC$ LENSIKDVII NQKITDKVDN LNQAVSVAKA TGDFSGVEQA LADLKNFSKG QLAQQAQKNE DFNTGKNSEL
$AB^{T}CCC$ LENSIKDVII NQKITDKVDN LNQAVSVAKA TGDFSGVEQA LADLKNFSKG QLAQQAQKNE DFNTGKNSEL
$AB^{T}CCCC$ LENSIKDVII NQKITDKVDN LNQAVSVAKA TGDFSGVEQA LADLKNFSKG QLAQQAQKNE DFNTGKNSEL
$AB^{T}D$ LENSIKDVII NQKITDKVDN LNQAVSVAKA TGDFSGVEQA LADLKNFSKG QLAQQAQKNE DFNTGKNSEL
WT G27 LENSVKDVII NQKVTDKVDN LNQAVSVAKA TGDFSRVEQA LADLKNFSKE QLAQQAQKNE DFNTGKNSAL
7.13 LENSIKDVII NQKITDKVDN LNQAVSVAKA TGDFSGVEQA LADLKNFSKG QLAQQAQKNE DFNTGKNSEL
841
AB^{T} YQSVKNGVNG TLVGNGLSQA EATTLSKNFS DIKKELNEKF KNFNNNNNNG LKN--EPIYA KVNKKKIGEV
$AB^{T}C$ YQSVKNGVNG TLVGNGLSQA EATTLSKNFS DIKKELNEKF KNFNNNNNNG LKN--EPIYA KVNKKKIGEV
$AB^{T}CC$ YQSVKNGVNG TLVGNGLSQA EATTLSKNFS DIKKELNEKF KNFNNNNNNG LKN--EPIYA KVNKKKIGEV
$AB^{T}CCC$ YQSVKNGVNG TLVGNGLSQA EATTLSKNFS DIKKELNEKF KNFNNNNNNG LKN--EPIYA KVNKKKIGEV
$AB^{T}CCCC$ YQSVKNGVNG TLVGNGLSQA EATTLSKNFS DIKKELNEKF KNFNNNNNNG LKN--EPIYA KVNKKKIGEV
$AB^{T}D$ YQSVKNGVNG TLVGNGLSKT EATTLTKNFS DIRKELNEKL FGNSNNNNNG LKNNTEPIYA QVNKKKAGQV
WT G27 YQSVKNGVNG TLVGNGLSKA EATTLSKNFS DIKKELNAKL GNFNNNNNNG LKNSTEPIYA KVNKKKAGQA
7.13 YQSVKNGVNG TLVGNGLSGI EATALTKNFS DIKKELNEKF KNFNNNNN-G LKNSGEPIYA QVNKKKTGQV
911
AB^{T} ASPEEPIYTQ VAKKVKAKID RLDQIASGLG DVGQATGFPL KRHTKVDD-- ---------- ----------
$AB^{T}C$ ASPEEPIYTQ VAKKVKAKID RLDQIASGLG DVGQATGFPL KRHTKVDDLS KVGLSANHEP IYATIDDLGG
$AB^{T}CC$ ASPEEPIYTQ VAKKVKAKID RLDQIASGLG DVGQATGFPL KRHTKVDDLS KVGLSANHEP IYATIDDLGG
$AB^{T}CCC$ ASPEEPIYTQ VAKKVKAKID RLDQIASGLG DVGQATGFPL KRHTKVDDLS KVGLSANHEP IYATIDDLGG
$AB^{T}CCCC$ ASPEEPIYTQ VAKKVKAKID RLDQIASGLG DVGQATGFPL KRHTKVDDLS KVGLSANHEP IYATIDDLGG
$AB^{T}D$ ASPEEPIYTQ VAKKVSAKID QLNEATSAIN RKIDRINKIA SAGKGVGAFS GAGRSASPEP IYATIDFDEA
WT G27 ASPEEPIYAQ VAKKVNAKID RLNQIASGLG VVGQAVGFPL KRHDKVGDLS KVGQSVSPEP IYATIDDLGG
7.13 ASPEEPIYTQ VAKKVKAKID QFNQVASGLG GVGQA-GFSL KGHTKVDDLS KVGRSVSPEP IYATIDDLGG

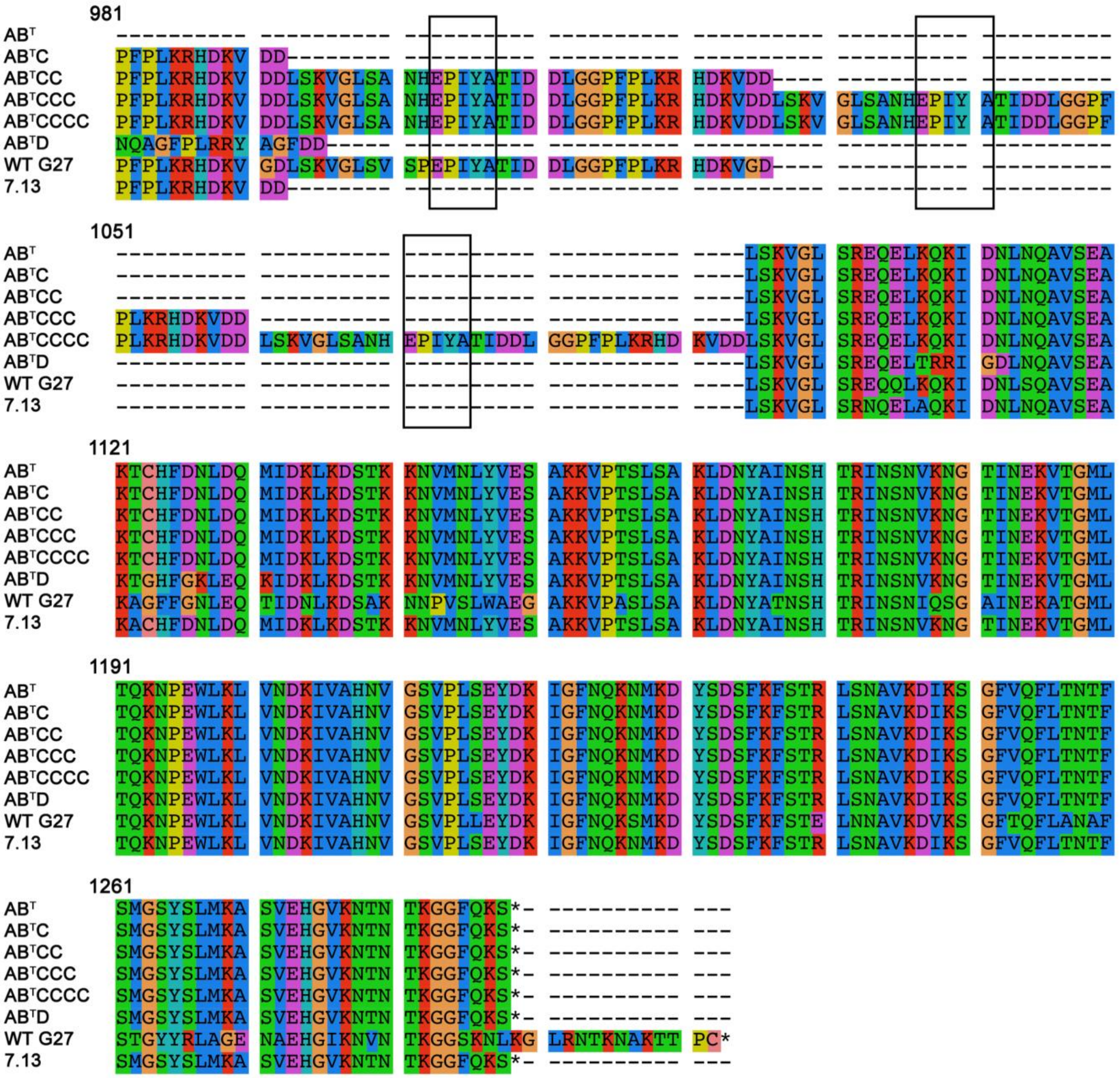

Supplementary Figure S1. CagA Isogenic Strain Protein Sequence Alignment. A protein alignment of CagA from the AB^T, AB^{T}C, AB^{T}CC, AB^{T}CCC, AB^{T}CCCC, and AB^{T}D isogenic strains is shown in comparison to WT G27 (HPG27_RS02655) and 7.13 (VD16_RS00005); strain 7.13 served as the template for the EPIYA motif flanking regions. The EPIYA and EPIYT motifs are denoted by boxes.

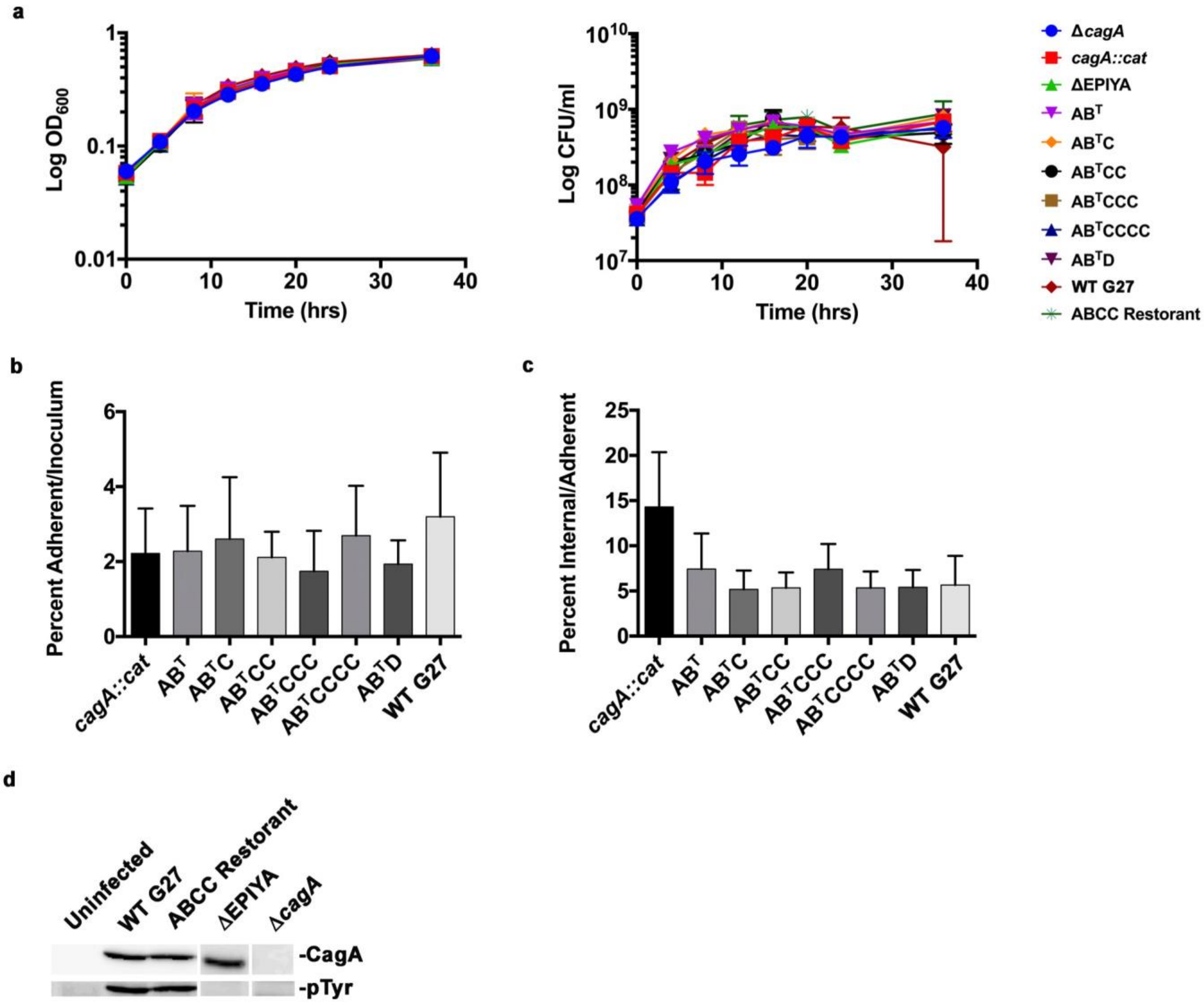

Supplementary Figure S2. CagA Isogenic Strain Characterization. (**a**). To monitor bacterial growth, 0.05 OD_{600} controlled cultures of the G27 isogenic strains were monitored every 4 hrs for 36 hrs and at 48 hrs. At each time point, an aliquot of culture was removed and growth was assessed by measuring the OD_{600} and by serial dilution and plating (colony forming units (CFU)/mL). Data are presented as the mean and range of two independent experiments. The data show that alteration of the CagA EPIYA region does not alter bacterial growth as compared to WT G27. (**b-c**) To determine if CagA EPIYA polymorphism alters bacterial adherence or internalization, AGS cells were infected in quadruplicate with the isogenic strains at a multiplicity of infection (MOI) of 100 for 2 hrs. The cells were then washed five times to remove non-adherent *H. pylori*. (**b**) To monitor bacterial adherence relative to the

inoculum, two replicates of the cells were lysed, serially diluted, and plated for CFU. (**c**) Bacterial internalization was monitored by treating the remaining two replicates of cells with 200 μg/mL gentamicin (gent) for 3 hrs, lysis, serially dilution, and plating. The percent of adherent *H. pylori* was determined relative to the starting inoculum. The percent of internalized *H. pylori* was determined relative to the number of adherent *H. pylori*. There were no statistically significant differences between strains in adherence to (*P*=0.9932) and internalization into AGS (*P*=0.5306) as analyzed by an ordinary one-way ANOVA. The data are presented as the mean + the SEM and represent three independent experiments. Together the data show that CagA EPIYA polymorphism does not alter bacterial growth or adherence to or internalization into host cells. (**d**). To characterize CagA translocation and phosphorylation following strain construction, AGS cells were infected for 8 hrs at an MOI of 100 with WT G27 or with the ABCC Restorant, ΔEPIYA, and Δ*cagA* control strains. Lysates were analyzed for total CagA (CagA) and phosphorylated CagA (pTyr). The Western blot images were cropped to show only the region corresponding to CagA (top) and phosphorylated CagA (bottom). As expected, no CagA was detected in the uninfected cells or the Δ*cagA* control infected lysates. No phosphorylated band for CagA was detected in the ΔEPIYA or Δ*cagA* infected lysates.

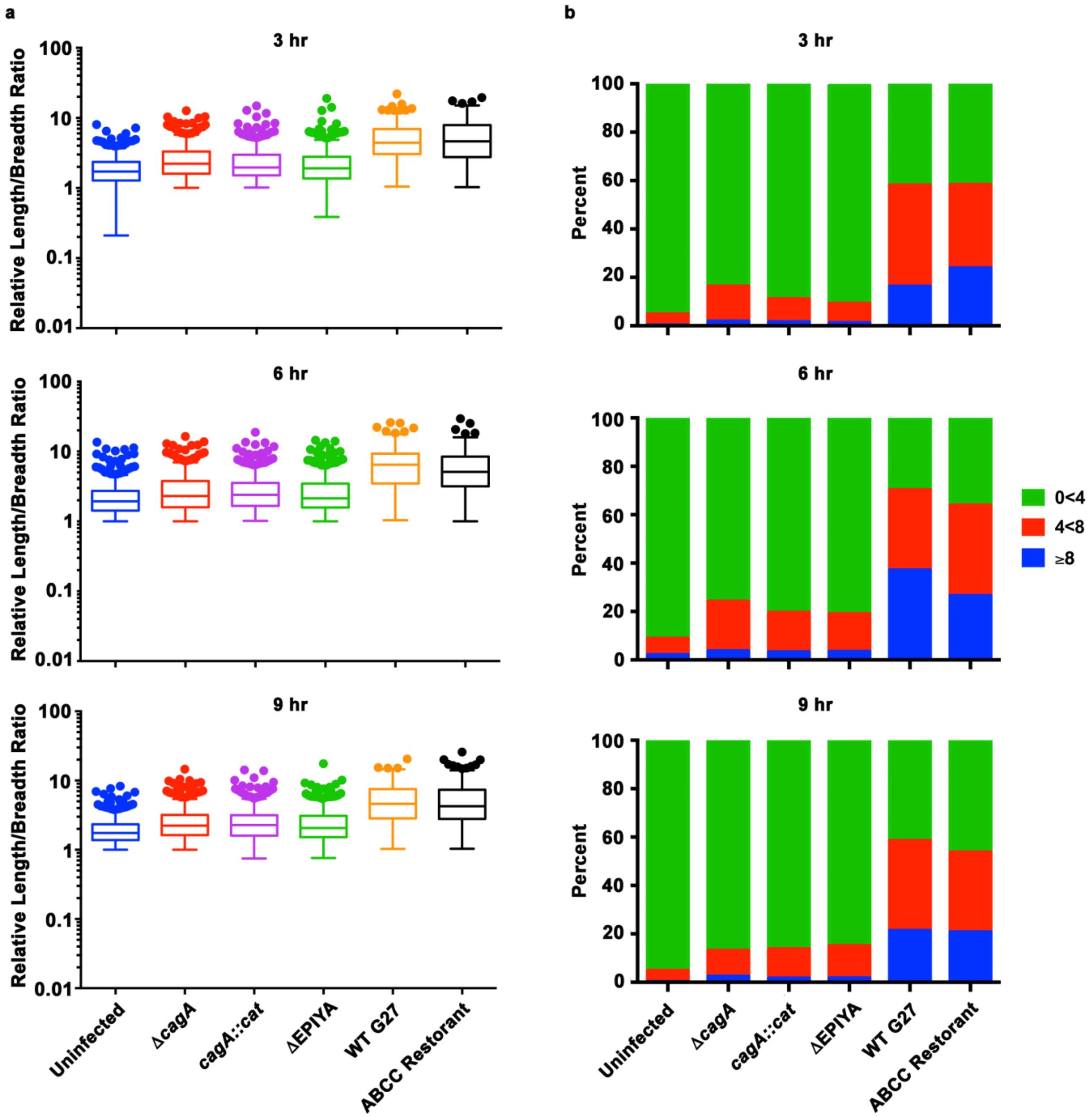

Supplementary Figure S3. AGS cell elongation induced by control strains. AGS cells were infected with the control panel of isogenic strains at an MOI of 100 for 3, 6, or 9 hrs. The cells were fixed and imaged by DIC microscopy. The relative length/breadth ratio was determined based on the maximum length of the cell divided by the maximum width (breadth) of the cell (modified from [1]). (**a**) The relative length/breadth ratios for each time point were graphed using a Tukey's box and whisker plot to show the distribution of ratios across time. Ratios that were greater than the 75th percentile + the

1.5 IQR are shown as individual data points. Differences in host cell elongation between strains were determined by an ordinary one-way ANOVA (see Supplementary Table S2). (**b**) The relative length/breadth ratios were then grouped (0<4, 4<8, ≥8) and the percent of elongated cells was determined to better illustrate changes in elongation across strains. AGS cell elongation was significantly increased for infected cells compared to the uninfected controls, and for cells infected with an intact EPIYA region (G27 and ABCC Restorant) as compared to Δ*cagA*, ΔEPIYA, and *cagA::cat*. There were no appreciable differences in elongation between the Δ*cagA*, ΔEPIYA, and *cagA::cat* strains or between the G27 and the ABCC Restorant strains. The data represent three independent experiments.

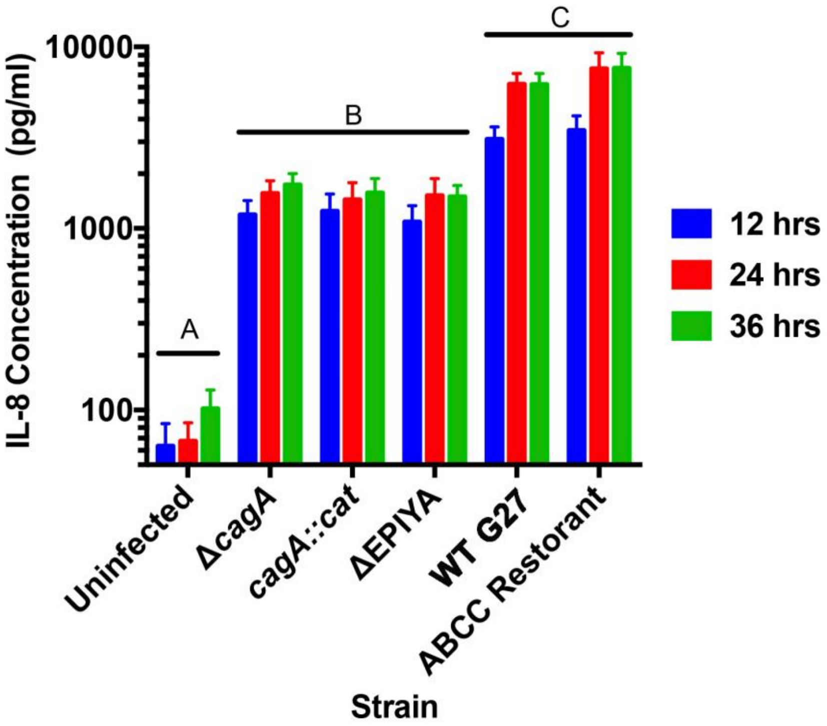

Supplementary Figure S4. IL-8 secretion is infection and EPIYA dependent. AGS cells were infected with the isogenic control strains at an MOI of 10. A sample of the co-culture supernatant was taken at 12, 24, and 36 hrs post-infection and analyzed by ELISA for total IL-8. At all time points, there was a significant increase in IL-8 secretion in cells infected with *H. pylori* as compared to uninfected control cells (Groups B and C vs. A; $P<0.0001$, two-way ANOVA). There was a significant increase in IL-8 secretion from cells infected with *H. pylori* that expressed CagA with an intact EPIYA motif (Group C) as compared to strains missing or containing a truncated CagA (Group B; $P<0.05$, two-way ANOVA). There were no differences in IL-8 secretion between cells infected with Δ*cagA*, ΔEPIYA, and *cagA::cat* or between WT G27 and the ABCC Restorant. ELISA data are presented as the geometric mean + 95% confidence interval and represent three independent experiments. *P* values were adjusted for multiple comparisons using the Tukey's multiple comparison test.

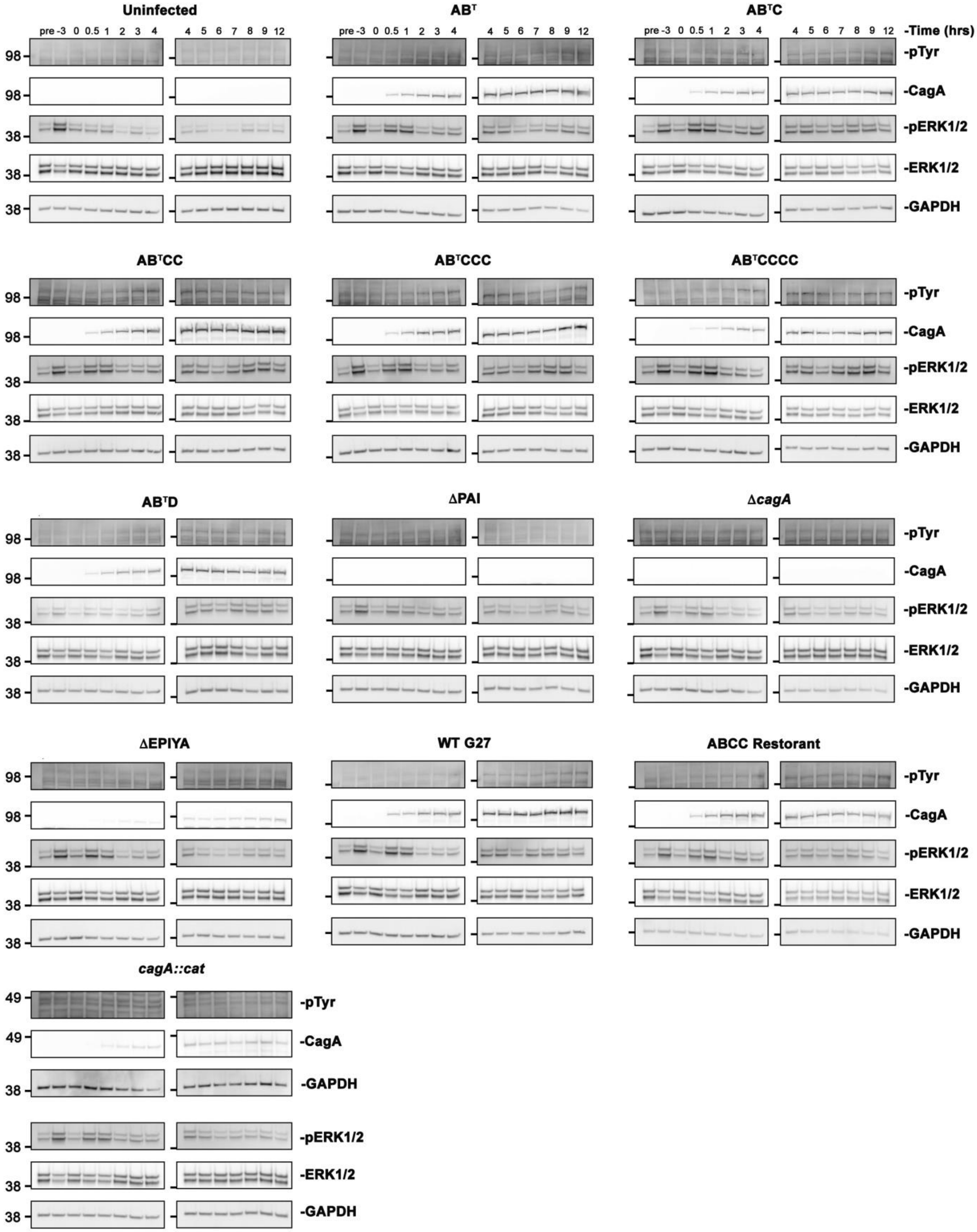
Uninfected
AB^T
AB^TC
-Time (hrs)
pre -3 0 0.5 1 2 3 4
4 5 6 7 8 9 12
98
38
-pTyr
-CagA
-pERK1/2
-ERK1/2
-GAPDH
AB^TCC
AB^TCCC
AB^TCCCC
AB^TD
ΔPAI
ΔcagA
ΔEPIYA
WT G27
ABCC Restorant
cagA::cat
49

Supplementary Figure S5. Western blot Analysis of CagA translocation, phosphorylation, and activation of ERK1/2. A set of AGS cells was harvested to determine a baseline reading of host cell pathway activation (time: pre). The cells were then washed and the medium replaced with medium supplemented with 2% fetal bovine serum (FBS) and a set of samples was harvested to determine activation of host cell pathways due to the media change (time: -3 hrs). The AGS cells were then incubated for 3 hrs to facilitate equilibration back to baseline prior to infection. Another set of samples was harvested immediately prior to infection (time: 0 hrs). AGS cells were then infected at an MOI of 100 and samples then harvested at the indicated time points (time: 0.5-12 hrs). Harvested lysates were analyzed by SDS-PAGE and Western blot for phosphorylated CagA (pTyr), total CagA (CagA), phosphorylated ERK1/2 (pERK1/2), and total ERK1/2 (ERK1/2). GAPDH served as a loading control. Due to the number of samples harvested across the time course for each strain or uninfected controls, the samples for each strain or the uninfected controls were run on two sets of blots; the 4 hr sample for each condition was run on both sets of blots for normalization and quantitation across blots. This Western blot data is representative of four independent experiments was cropped to show the indicated proteins.

.

SUPPLEMENTARY METHODS

Isogenic strain construction.

G27 ΔEPIYA. The G27 ΔEPIYA strain was constructed so that the C-terminal EPIYA region of the G27 wild type (WT) strain was replaced with a counter-selectable kanamycin resistance determinant *aphA3/sacB* (*kan-sacB*) [2,3], which encodes kanamycin resistance and sucrose sensitivity. This strain served as the parental strain for the CagA isogenic strain construction. Briefly, the N-terminal and C-terminal regions flanking the *H. pylori* 7.13 *cagA* EPIYA motifs were PCR amplified using 7.13 genomic DNA (gDNA) as a template and the 7.13ΔEPIYA-5'-fp/7.13ΔEPIYA-Mrp and 7.13ΔEPIYA-Mfp/7.13ΔEPIYA-3'-rp primers, respectively. The 7.13ΔEPIYA-Mrp and 7.13ΔEPIYA-Mfp primers were engineered to contain XhoI and SmaI restriction sites. The resulting PCR fragments were fused by SOE PCR using 7.13ΔEPIYA-5'-fp/7.13ΔEPIYA-3'-rp. The SOE product was cloned into pGEM-T Easy to yield pDSM530. EcoR1, XhoI, and SmaI digestion and sequencing with T7/SP6 primers were used to verify proper insertion of the SOE product into the vector. pDSM530 and pKSF-II were next individually digested with XhoI and SmaI and the liberated *kan-sacB* cassette from pKSF-II was ligated into pDSM530 to yield pDSM531. *E. coli* transformants were selected for by growth on kanamycin and the resulting plasmid was verified by EcoR1 and Sma1 digestion. *H. pylori* G27 was then naturally transformed with pDSM531 and transformants were selected for on kanamycin. Proper double homologous recombination and insertion of the *kan-sacB* cassette into the G27 genome was verified by PCR with the G27ΔEPIYA-5'-fp/7.13ΔEPIYA-Mrp and SacBSCN-F2/Grace2 primer sets. The resulting G27 ΔEPIYA strain was archived as DSM713.

CagA EPIYA-AB^{T}, -AB^{T}C, -AB^{T}CC, -AB^{T}CCC, and -AB^{T}CCCC Constructs. To create the EPIYA-AB^{T}, -AB^{T}C, -AB^{T}CC, -AB^{T}CCC, and -AB^{T}CCCC CagA variants, the CagA EPIYA-AB^{T}CCCC region from the Korean isolate K154 (DSM591) [4] was used. Of note, the EPIYA-B motif in this strain contains a natural alanine to threonine amino acid change (EPIYT) and is denoted as B^{T} [4]. Briefly, the EPIYA-AB^{T} strain was created through a series of PCR reactions that amplified the flanking regions from 7.13 *cagA* and K154 AB^{T}. To obtain the products for SOE, four PCR reactions were conducted as follows: 1) primer pair 7.13ΔEPIYA-5'-fp/K154-7IS-M1rp was used with 7.13 gDNA to amplify the *cagA*

upstream flanking region; 2) primer pair K154-7IS-M1fp/K154-7IS-M12rp was used with K154 gDNA to amplify the AB^{T} motif; 3) primer pair K154-7IS-M12fp/K154-7IS-M2rp was used with K154 gDNA to amplify the region immediately downstream of the EPIYA motifs; and 4) primer pair K154-7IS-M2fp/7.13ΔEPIYA-3'-rp was used with 7.13 gDNA to amplify the downstream flanking region. Next, these products were purified and mixed for SOE PCR. The first SOE PCR was performed using the primer pair 7.13ΔEPIYA-5'-fp and K154-7IS-M12rp to join the PCR products from the first and second PCR reactions. The second SOE PCR joined the third and fourth PCR products using K154-7IS-M12fp and 7.13ΔEPIYA-3'-rp. A third SOE PCR joined the products from the two SOE PCR reactions using 7.13ΔEPIYA-5'-fp and 7.13ΔEPIYA-3'-rp. This final product was cloned into pGEM-T Easy to generate pDSM532. Proper insertion was verified by EcoRI and SmaI digestion and sequencing using the T7/SP6 and K154-7IS-M1fp/K154-7IS-M2rp primers. G27ΔEPIYA was then naturally transformed with pDSM532; insertion of the EPIYA-AB^{T} construct by double homologous recombination was screened for by loss of the *kan-sacB* cassette as indicated by sucrose resistance and kanamycin sensitivity. gDNA of the transformants was screened by PCR using primers K154-7IS-M1fp/K154-7IS-M2rp to identify the proper sized product. Following expansion of the proper transformant, gDNA was isolated and the *cagA* EPIYA flanking regions were PCR amplified using the primers G27ΔEPIYA-5'fp/K154-7IS-M1rp and K154-7IS-M2fp/Grace2. The PCR product was sequenced with K154-7IS-M1rp and K154-7IS-M2fp primers. The resulting strain was archived as DSM714.

The CagA EPIYA-AB^{T}C, -AB^{T}CC, -AB^{T}CCC, -AB^{T}CCCC constructs resulted from a series of PCR reactions that yielded ladder PCR bands that corresponded to each of the desired constructs. The PCR reactions were as follows: 1) 7.13 gDNA was amplified with 7.13ΔEPIYA-5'-fp/K154-7IS-M1rp for the upstream flanking region; 2) K154 gDNA was amplified with K154-7IS-M1fp/K154-7IS-M2rp for the EPIYA motifs; and 3) 7.13 gDNA was amplified with K154-7IS-M2fp/7.13ΔEPIYA-3'-rp for the downstream flanking region. As above, the products were purified and a series of three SOE PCR reactions were performed. First, the first and second PCR products were joined with the 7.13ΔEPIYA-5'-fp/K154-7IS-M2rp primers. Next, the second and third PCR products were joined using the K154-7IS-M1fp/7.13ΔEPIYA-3'-rp primers. Finally, the first and second SOE PCR products were joined with

7.13ΔEPIYA-5'-fp/7.13ΔEPIYA-3'-rp primers. The ladder PCR bands were individually isolated and cloned into pGem-T Easy to yield the following: pDSM570 for the EPIYA-AB^{T}C construct, pDSM571 for the EPIYA-AB^{T}CC construct, pDSM572 for the AB^{T}CCC construct, and pDSM573 for the EPIYA-AB^{T}CCCC construct. Each construct was verified by EcoRI and SmaI digestion and sequencing with T7/SP6 primers and K154-7IS-M1fp/K154-7IS-M2rp primers. Each plasmid was then independently transformed into the G27 ΔEPIYA strain, and transformants were selected for by sucrose resistance and kanamycin sensitivity as described above. To screen for the loss of the *kan-sacB* cassette, the *cagA* EPIYA flanking region was PCR amplified from the gDNA of each transformant using the primers K154-7IS-M1fp/K154-7IS-M2rp to identify the proper sized product. Following expansion, gDNA was isolated from the transformants and the *cagA* EPIYA flanking regions were PCR amplified using the G27ΔEPIYA-5'fp/K154-7IS-M1rp and K154-7IS-M2fp/Grace2. The PCR product was then sequenced with K154-7IS-M1rp and K154-7IS-M2fp. The resulting strains were archived as DSM715 for EPIYA-AB^{T}C, DSM716 for -AB^{T}CC, DSM717 for -AB^{T}CCC, and DSM718 for -AB^{T}CCCC.

CagA EPIYA-AB^{T}D Construct. The construction of the CagA EPIYA-AB^{T}D strain was completed in two parts. First, the ABD construct was PCR amplified in a series of reactions from the clinical Korean strain K3 (DSM590) [4]. The PCR reactions were as follows: 1). 7.13 gDNA was amplified with 7.13ΔEPIYA-5'-fp/K3-7IS-M1rp for the upstream flanking region; 2) K3 gDNA was amplified with K3-7IS-M1fp/K3-7IS-M2rp for the EPIYA motifs; and 3) 7.13 gDNA was amplified with K3-7IS-M2fp/7.13ΔEPIYA-3'-rp for the downstream flanking region. As previously described, the products were joined by SOE PCR such that the first and second PCR products were joined with 7.13ΔEPIYA-5'-fp/K3-7IS-M2rp; this product was then joined with the third PCR product using the 7.13ΔEPIYA-5'-fp/7.13ΔEPIYA-3'-rp primer pair. The ABD PCR product was cloned into pGEM-T EASY to yield pDSM533; proper insertion and sequencing with T7/SP6 were performed as described above. In the second phase of construction, site directed mutagenesis was performed on pDSM533 in a series of three PCR reactions to change the alanine in the EPIYA-B motif to a threonine to yield an EPIYT motif (denoted as-B^{T}). The PCR reactions using pDSM533 were as follows: 1) 7.13ΔEPIYA-5'-fp and K3(A/T)-rp; and 2) K3(A/T)-fp and 7.13ΔEPIYA-3'-rp. Next, these PCR products were purified, mixed

and joined by SOE PCR with the 7.13ΔEPIYA-5'-fp/7.13ΔEPIYA-3'-rp primer pair. The SOE product was cloned into pGEM-T Easy to yield pDSM547 and the site directed mutagenesis was verified by sequencing with T7/SP6 primers. G27 ΔEPIYA was naturally transformed with pDSM547 and proper insertion of the EPIYA-AB^{T}D construct was verified by sucrose resistance and kanamycin sensitivity. gDNA from the transformants was screened by PCR using primers K154-7IS-M1fp/K154-7IS-M2rp to identify the proper sized product. Following expansion of the correct transformant, gDNA was isolated from the transformant and the *cagA* EPIYA flanking regions were PCR amplified using the G27ΔEPIYA-5'fp/K154-7IS-M1rp and K154-7IS-M2fp/Grace2 primers. The PCR product was then sequenced using the K154-7IS-M1rp/K154-7IS-M2fp primers. The resulting strain was archived as DSM719

G27 Δ*cagA*. The G27 Δ*cagA* strain was constructed such that the entire *cagA* coding sequence was replaced with the *kan-sacB* counter-selectable cassette. To accomplish this, primer pairs G27ΔCagAfp/G27ΔCagA-Mrp and G27ΔCagArp/G27ΔCagA-Mfp, were used to PCR amplify the N-terminal and C-terminal flanking regions of the G27 *cagA* coding sequence, respectively. G27ΔCagA-Mrp and G27ΔCagA-Mfp were engineered to include XhoI and SmaI restriction sites for cloning of the *kan-sacB* cassette. The two PCR fragments were combined by SOE amplification with the G27ΔCagAfp and G27ΔCagArp primers, and the subsequent fragment was cloned into pGEM-T Easy (pDSM1389). Proper insertion of the SOE product was verified with EcoRI, XhoI, and SmaI digestion and by sequencing with the T7/SP6 primers. As described for the ΔEPIYA strain, the pDSM1389 and pKSF-II plasmids were digested with SmaI and XhoI in a sequential reaction prior to ligation of pDSM1389 with the liberated *kan-sacB* cassette. pDSM1390 was then naturally transformed and integrated into G27 by double homologous recombination. Insertion of the *kan-sacB* cassette into the *cagA* locus was verified by kanamycin resistance and sucrose sensitivity and by PCR using the G27ΔCagAfp/G27ΔCagArp, SacBSCN-F2/Grace1, and SacBSCN-F2/Grace2 primer pairs for the proper sized products. The resulting strain was archived as DSM1391.

G27 ABCC Restorant. As a control for genetic manipulation of the strains, the G27 ΔEPIYA strain was restored to the WT ABCC EPIYA sequence. The ABCC EPIYA region was PCR amplified from G27 gDNA with G27EPIYA-F/G27EPIYA-R. The resulting PCR product was cloned into pGEM-T

Easy to yield pDSM1419; proper sequence was verified by using the T7/SP6 sequencing primers in combination with primers that aligned to various regions of the *cagA* EPIYA sequence (CagA2864R, CagA3149F, CagA3493R, and CagA3576F). The G27 ΔEPIYA strain was then transformed with pDSM1419. Transformants were screened for by loss of kanamycin resistance and sucrose sensitivity. Proper insertion of the EPIYA region in the transformants was verified by PCR amplification of the region using the G27EPIYASeq-F/Grace2 primers to screen for the correct sized product. The PCR product was then sequenced with the G27EPIYASeqF, CagA3149F, G27EPIYA-R, and Grace1 primers to confirm replacement of the *kan-sacB* cassette with the WT ABCC EPIYA sequence. The resulting strain was archived as DSM1420.

G27 *cagA::cat*. G27-MA Δ*cagA* (DSM205) was previously constructed [5] and contains a chloramphenicol acetyltransferase (*cat*) cassette that disrupts the *cagA* gene. The mutant construct was PCR amplified from this strain with the cagA-F(-13) and cag seq 6 primers using the Expand High Fidelity-Plus System (Roche Diagnostics, Indianapolis, IN). The resulting 3 kb fragment was purified and ligated into pGEM-T Easy to yield pDSM729. The *E. coli* transformants were selected for by chloramphenicol resistance and verified by EcoRI digestion. WT G27 was naturally transformed with pDSM729 and *H. pylori* transformants were selected on chloramphenicol. gDNA isolated from the resulting transformants were verified by PCR using the cagA-F(-13)/cag seq 12 and cagA-F(-13)/cag seq 8 primer pairs for appropriately sized products. The resulting strain was archived as DSM1420.

G27 ΔPAI. G27-MA ΔPAI (DSM206) was previously constructed [5] and contains a kanamycin cassette instead of the PAI. The mutant construct was PCR amplified from this strain with the ΔPAI-F and Grace1 primers using the Expand High Fidelity-Plus System (Roche Diagnostics). The resulting 3kb fragment was purified and ligated into pGEM-T Easy (pDSM730). *E. coli* transformants were selected for by kanamycin resistance and verified by EcoRI digestion. WT G27 was then transformed with pDSM730 and transformants were selected for by kanamycin resistance. gDNA isolated from the resulting transformants was analyzed using the primer pairs ΔPAI-F/Grace1 and cagA-F(-13)/cag seq 2 to verify proper insertion of the kanamycin cassette based on the appropriately sized products. The resulting strain was archived as DSM723.

Bacterial growth analysis. To monitor the growth characteristics of the isogenic strains, 30 mL 0.05 ODU controlled liquid cultures were sampled every 4 hrs for 24 hrs and again at 36 hrs. At each time point, an aliquot of the culture was removed and the OD_{600} was recorded. The culture was also plated on HBA plates to determine CFU. The plates were incubated for 4 - 5 days under microaerobic conditions and the CFU enumerated.

Bacterial Adherence to and Internalization into AGS cells. AGS cells were seeded at 2.0 - 2.2 x 10^5 in 24-well tissue culture plates 18-24 hrs prior to infection. Before infection, cells were washed with phosphate buffered saline (PBS) and medium was replaced with F-12K supplemented with 10% FBS, 10% Brucella broth, and 10 μg/ml vancomycin. AGS cells were then infected in quadruplicate with the isogenic strains at a MOI of 100 for 2 hrs. Following infection, cells were washed five times with PBS to remove non-adherent *H. pylori*. To monitor bacterial adherence, two infection replicates were lysed with 1% saponin in PBS, serially diluted, and plated. For bacterial internalization, two infection replicates were incubated with F12K supplemented with 200 μg/mL gent for 3 hr, lysed with 1% saponin in PBS, serially diluted, and plated. Plates were incubated for 4 - 5 days under microaerobic conditions, the CFU were enumerated, and the data from technical replicates was averaged. The percent of adherent *H. pylori* was determined relative to the starting inoculum. The percent of internalized *H. pylori* was determined relative to the number of adherent *H. pylori*. Presented data were obtained from three independent experiments.

Supplementary Table S1. ***P*** **values* for EPIYA motif induced AGS cell elongation.**

Strain	3 hr	6 hr	9 hr
Uninfected vs. *cagA::cat*	0.1989	0.5638	0.9788
Uninfected vs. AB^T	<0.0001	<0.0001	<0.0001
Uninfected vs. AB^TC	<0.0001	<0.0001	<0.0001
Uninfected vs. AB^TCC	<0.0001	<0.0001	<0.0001
Uninfected vs. AB^TCCC	<0.0001	<0.0001	<0.0001
Uninfected vs. AB^TCCCC	<0.0001	<0.0001	<0.0001
Uninfected vs. AB^TD	<0.0001	<0.0001	<0.0001
Uninfected vs. WT G27	<0.0001	<0.0001	<0.0001
cagA::cat vs. AB^T	0.0001	<0.0001	<0.0001
cagA::cat vs. AB^TC	<0.0001	<0.0001	<0.0001
cagA::cat vs. AB^TCC	<0.0001	<0.0001	<0.0001
cagA::cat vs. AB^TCCC	<0.0001	<0.0001	<0.0001
cagA::cat vs. AB^TCCCC	<0.0001	<0.0001	<0.0001
cagA::cat vs. AB^TD	<0.0001	<0.0001	<0.0001
cagA::cat vs. WT G27	<0.0001	<0.0001	<0.0001
AB^T vs. AB^TC	<0.0001	<0.0001	0.0005
AB^T vs. AB^TCC	<0.0001	<0.0001	0.0910
AB^T vs. AB^TCCC	<0.0001	<0.0001	0.9775
AB^T vs. AB^TCCCC	<0.0001	<0.0001	0.1755
AB^T vs. AB^TD	<0.0001	<0.0001	0.9949
AB^T vs. WT G27	<0.0001	<0.0001	0.0173
AB^TC vs. AB^TCC	0.9793	>0.9999	0.8492
AB^TC vs. AB^TCCC	0.0016	0.9987	0.0390
AB^TC vs. AB^TCCCC	<0.0001	>0.9999	0.6690
AB^TC vs. AB^TD	0.0461	0.9998	0.0206
AB^TC vs. WT G27	>0.9999	0.9989	0.9859
AB^TCC vs. AB^TCCC	0.0569	0.9685	0.7170
AB^TCC vs. AB^TCCCC	0.0002	>0.9999	>0.9999
AB^TCC vs. AB^TD	0.4287	>0.9999	0.5747
AB^TCC vs. WT G27	0.9995	>0.9999	0.9998
AB^TCCC vs. AB^TCCCC	0.7868	0.9756	0.8670
AB^TCCC vs. AB^TD	0.9987	0.9479	>0.9999
AB^TCCC vs. WT G27	0.0118	0.8981	0.3575
AB^TCCCC vs. AB^TD	0.3989	>0.9999	0.7546
AB^TCCCC vs. WT G27	<0.0001	>0.9999	0.9957
AB^TD vs. WT G27	0.1602	>0.9999	0.2405

Differences in host cell elongation for each time point were evaluated using an ordinary one-way ANOVA; **P* values were adjusted for multiple comparisons using Tukey's multiple comparison test. Shading represents non-significant *P* values.

Supplementary Table S2. *P* values* for control strain induced AGS cell elongation

Strain	3 hr	6 hr	9 hr
Uninfected vs. Δ*cagA*	<0.0001	<0.0001	<0.0001
Uninfected vs. ΔEPIYA	0.0343	<0.0001	<0.0001
Uninfected vs. *cagA::cat*	0.0002	0.0029	<0.0001
Uninfected vs. WT G27	<0.0001	<0.0001	<0.0001
Uninfected vs. ABCC Restorant	<0.0001	<0.0001	<0.0001
Δ*cagA* vs. ΔEPIYA	0.0005	>0.9999	0.9994
Δ*cagA* vs. *cagA::cat*	0.0897	0.8794	0.6549
Δ*cagA* vs. WT G27	<0.0001	<0.0001	<0.0001
Δ*cagA* vs. ABCC Restorant	<0.0001	<0.0001	<0.0001
ΔEPIYA vs. *cagA::cat*	0.6446	0.7505	0.4306
ΔEPIYA vs. WT G27	<0.0001	<0.0001	<0.0001
ΔEPIYA vs. ABCC Restorant	<0.0001	<0.0001	<0.0001
cagA::cat vs. WT G27	<0.0001	<0.0001	<0.0001
cagA::cat vs. ABCC Restorant	<0.0001	<0.0001	<0.0001
WT G27 vs. ABCC Restorant	0.9785	0.0222	0.9994

Differences in host cell elongation for each time point were evaluated using an ordinary one-way ANOVA; **P* values were adjusted for multiple comparisons using Tukey's multiple comparison test. Shading represents non-significant *P* values.

Supplementary Table S3. Primers used in this study.

**Primers**	**Description** [a]	**Reference**
**Cloning primers**		
7.13ΔEPIYA-5'-fp:	GTCTGATAAGTTTGAAAACATC	This study
7.13ΔEPIYA-Mrp (Xho1, SmaI)[b]	GTCTATCCCCGGG**AGG**CTCGAGCCCATTACCGACTAGGGTTCC	This study
7.13ΔEPIYA-Mfp: (Xho1, SmaI)	GTAATGGGCTCGAG**CCT**CCCGGGGATAGACAAGCTCAAAGATTC	This study
7.13ΔEPIYA-3'-rp:	CCTTGTTTTTAGCAAGGGGTGG	This study
cag seq 2:	CCCAAACCACTTGCTATTTG	This study
cag seq 6:	GCTTCAGCTACAGCTTTATTGA	This study
cag seq 8:	TGCAAGAAATTCCATGAAATC	This study
cag seq 12:	GGGTTGTATGATATTTTCC	This study
CagA2864R:	CGGAATTTTCAAGGTCGC	This study
CagA3149F:	CGGACATCAAGAAAGAGTTGAATGC	This study
CagA3493R:	CAAAGGGAAAGGTCCGCCG	This study
CagA3576F:	CGGCGGACCTTTCCCTTTG	This study
CagA-F(-13):	GTAAGGAGAAACAATGACTAACG	This study
ΔPAI-F	CCAAATTTTATAGGATTCGCGCTC	This study
G27Δ*cagA*fp:	CCATTTTAAGCAACTCCATAGACC	This study
G27Δ*cagA*-Mfp: (Xho1, SmaI)	GAAACAATGCTCGAG**CCT**CCCGGGCCAAAAATCTTAAAGGATTAAGG	This study
G27Δ*cagA*-Mrp: (Xho1, SmaI)	GATTTTTGGCCCGGG**AGG**CTCGAGCATTGTTTCTCCTTACTATACC	This study
G27Δ*cagA*rp:	CCATAAGTTTTCACGGTGG	This study
G27EPIYA-F:	GGGAATTGTCTGATAAACTTGAGAATG	This study
G27EPIYA-R	CCTTGTTTTTAGCAAGGGGTGG	This study
G27EPIYASeq-F	GGAAGCAAAATCTCAAGCTAACAGC	This study
G27ΔEPIYA-5'-fp:	GGCATCAAAAGGGAATTGTCTG	This study
K3-7IS-M1fp	GGAACCCTAGTCGGTAATGGGTTATCTAAAACAGAAGCCACAACG	This study
K3-7IS-M1rp	CGTTGTGGCTTCTGTTTTAGATAACCCATTACCGACTAGGGTTCC	This study
K3-7IS-M2fp	GGTCATTTTGGCAAACTAGAACAAAAGATAGACAAGCTCAAAGATTC	This study
K3-7IS-M2rp	GAATCTTTGAGCTTGTCTATCTTTTGTTCTAGTTTGCCAAAATGACC	This study
K154-7IS-M1fp:	GGAACCCTAGTCGGTAATGGGTTATCTCAAGCAGAAGCC	This study
K154-7IS-M1rp:	GGCTTCTGCTTGAGATAACCCATTACCGACTAGGGTTCC	This study
K154-7IS-M12fp:	GGGCTTTCAAGGGAGCAAGAATTGAAAC	This study
K154-7IS-M12rp:	GTTTCAATTCTTGCTCCCTTGAAAGCCCTACCTTACTGAG	This study
K154-7IS-M2fp:	GATAGACAAGCTCAAAGATTCTAC	This study
K154-7IS-M2rp:	GTAGAATCTTTGAGCTTGTCTATC	This study
K3(A/T)-fp (41mer)[c]	GCCCTGAAGAGCCCATTTACACTCAAGTTGCTAAAAAGGTG	This study

K3(A/T)-rp (41mer)[c]	CACCTTTTTAGCAACTTGAGTGTAAATGGGCTCTTCAGGGC	This study
**Screening and sequencing primers**		
T7	GGGTTTTCCCAGTCACGA	Promega
SP6	GCACCCCAGGCTTTACAC	Promega
SacBSCN-F2	CGAATCGAATTCAGGAAC	[6]
Grace1	GGTTGCACGCATTTTCCC	This study
Grace2	TCATGCGAGCGGCGATGT	[4]

[a]Restriction endonuclease sites are underlined and linker bases are in bold type.

[b]Important restrictions sites are included in parentheses.

[c]Primers were used to change the alanine to a threonine in the EPIYA-B motif resulting in EPIYT

SUPPLEMENTARY REFERENCES

1 Bourzac, K. M., Botham, C. M. & Guillemin, K. *Helicobacter pylori* CagA induces AGS cell elongation through a cell retraction defect that is independent of Cdc42, Rac1, and Arp2/3. *Infect Immun* **75**, 1203-1213, doi:10.1128/IAI.01702-06 (2007).

2 Copass, M., Grandi, G. & Rappuoli, R. Introduction of unmarked mutations in the *Helicobacter pylori vacA* gene with a sucrose sensitivity marker. *Infect Immun* **65**, 1949-1952 (1997).

3 Mehta, N., Olson, J. W. & Maier, R. J. Characterization of *Helicobacter pylori* nickel metabolism accessory proteins needed for maturation of both urease and hydrogenase. *J Bacteriol* **185**, 726-734 (2003).

4 Jones, K. R. *et al.* Polymorphism in the CagA EPIYA motif impacts development of gastric cancer. *J Clin Microbiol* **47**, 959-968, doi:10.1128/JCM.02330-08 (2009).

5 Amieva, M. R., Salama, N. R., Tompkins, L. S. & Falkow, S. *Helicobacter pylori* enter and survive within multivesicular vacuoles of epithelial cells. *Cell Microbiol* **4**, 677-690 (2002).

6 Carpenter, B. M. *et al.* A single nucleotide change affects *fur*-dependent regulation of *sod*B in *H. pylori*. *PLoS One* **4**, e5369, doi:10.1371/journal.pone.0005369 (2009).
